# Impact of measurement noise, experimental design, and estimation methods on Modular Response Analysis based network reconstruction

**DOI:** 10.1038/s41598-018-34353-3

**Published:** 2018-11-01

**Authors:** Caterina Thomaseth, Dirk Fey, Tapesh Santra, Oleksii S. Rukhlenko, Nicole E. Radde, Boris N. Kholodenko

**Affiliations:** 1 University of Stuttgart, Institute for Systems Theory and Automatic Control, Stuttgart, Germany; 2 University College Dublin, Systems Biology Ireland, UCD School of Medicine, Dublin, Ireland; 3 Department of Pharmacology, Yale University School of Medicine, New Haven, CT, USA

## Abstract

Modular Response Analysis (MRA) is a method to reconstruct signalling networks from steady-state perturbation data which has frequently been used in different settings. Since these data are usually noisy due to multi-step measurement procedures and biological variability, it is important to investigate the effect of this noise onto network reconstruction. Here we present a systematic study to investigate propagation of noise from concentration measurements to network structures. Therefore, we design an *in silico* study of the MAPK and the p53 signalling pathways with realistic noise settings. We make use of statistical concepts and measures to evaluate accuracy and precision of individual inferred interactions and resulting network structures. Our results allow to derive clear recommendations to optimize the performance of MRA based network reconstruction: First, large perturbations are favorable in terms of accuracy even for models with non-linear steady-state response curves. Second, a single control measurement for different perturbation experiments seems to be sufficient for network reconstruction, and third, we recommend to execute the MRA workflow with the mean of different replicates for concentration measurements rather than using computationally more involved regression strategies.

## Introduction

**Figure 1:**
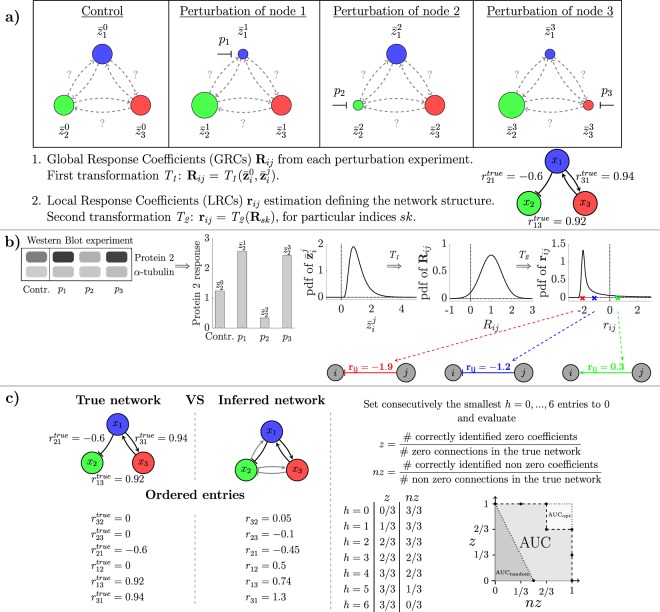
Variability and noise in measurements affect network reconstruction via Modular Response Analysis. **(a**) MRA workflow for a three-node network. After subsequent perturbation of all nodes and quantification of concentration fold changes, LRCs **r**_*ij*_ are calculated via a two-step non-linear transformation. **(b)** One exemplary realization (one replicate) of the noisy measurement for protein 2 is evaluated in all experimental conditions (left part). These values are interpreted as samples of the corresponding distributions, described by the probability density function (pdf of ${\bar{{\bf{z}}}}_{i}^{j}$) arising from the measurement noise. On the right we describe how network reconstruction is affected by the variability of the measured protein concentrations in terms of propagation of distributions from the measurements ${\bar{{\bf{z}}}}_{i}^{j}$ and ${\bar{{\bf{z}}}}_{i}^{0}$ via the GRCs **R**_*ij*_ to the LRCs **r**_*ij*_. (**c**) Performance evaluation of inferred network structures is done by using the assessment method presented in^9^, which compares the inferred structure with a reference structure. Similar to ROC analysis, the Area Under the Curve (AUC) serves as a normalized measure for the fit quality, and varies from the average value 0.25 in the random case (darker grey triangle) up to the optimal value 1 for a correctly identified network (whole square).

Advanced experimental techniques have facilitated our mechanistic understanding of intracellular processes in the last decades. However, the problem of network reconstruction from experimental data remains a challenging task, for which many different approaches have been suggested. Among those, Modular Response Analysis (MRA) has been proven successful in many applications^1–4^. MRA uses steady-state data of experiments in which each node of a network is perturbed successively (see Fig. 1a). These steady-state data are transformed into quantitative pairwise interaction strengths, denoted Local Response Coefficients (LRCs), which define the network structure. This is done in a two-step process, in which first concentration measurements are transformed into Global Response Coefficients (GRCs), which are then used to calculate the LRCs. MRA is an elegant method that gives reliable results in case that concentrations can be accurately measured and measurement noise can be neglected^5^.

MRA is however often applied in settings in which we have to deal with real noisy experimental data and few replicates, for example when using Western blotting to investigate signalling pathways, as exemplified in^1,2^. In these studies the authors addressed the issue of measurement noise by using statistical approaches, like Monte Carlo simulations or maximum likelihood, to estimate interaction strengths and respective uncertainties. Despite the extensive usage of MRA, the effect of noise in the input data on network reconstruction is not completely understood. Recent developments include statistical reformulations of the MRA that have been suggested to address the issue of noisy and sparse/insufficient data^6^. A further extension combines the classical deterministic MRA framework with advanced nonparametric single-cell data resampling to discriminate between direct and indirect connectivities^7^.

Despite the broad literature tackling the issue of experimental noise, a comprehensive and systematic study on how network inference via MRA is affected by noise is still required. In particular, some of the studies do not consider noise propagation from the input data to the estimated LRCs, but start directly with the GRCs^8^. Furthermore, a realistic statistical characterization of the MRA variables (measured data, GRCs and LRCs) and a robustness analysis in a general experimental setup are also still missing.

In this study we develop a statistical framework to analyse noise propagation by transformations of input into output distributions (Fig. 1b). By comparing different experimental and computational strategies in an *in silico* study, we derive recipes for experimentalists and modellers regarding an optimal MRA workflow design. In particular, we investigate (1) how non-linear transformations and mathematical approximations of the MRA framework affect noise propagation; (2) the influence of perturbation strength, control strategy and number of replicates on the uncertainty of the estimated interactions; (3) the effects of different estimation methods on the performance of the network inference problem.

To evaluate the resulting network structure, we apply a performance evaluation method that was proposed in^9^ and is schematically depicted in Fig. 1c. It works similar to a Receiver Operating Characteristic (ROC) and its Area Under the Curve (AUC value) for evaluating the performance of a classifier but additionally takes correctness of the sign of an inferred interaction into account. A correctly identified network has an AUC value of 1 (whole square), the random case corresponds on average to an AUC value of 0.25 (darker grey triangle).

Results are given for two test-bed examples of well-known signalling pathways, a model for the mitogen-activated protein kinase (MAPK) pathway and a model for the tumour suppressor protein p53. These models show very different non-linear properties regarding their steady-state behaviour in dependence of perturbation strengths. Our results show that large perturbations and few technical replicates, combined with a simple control strategy and a basic estimation method, lead to an optimal bias-variability trade-off of the estimated pairwise interactions and also give robust results regarding network reconstruction.

## Results

### MAPK and p53 test-bed models with complementary dynamic behaviours

**Figure 2:**
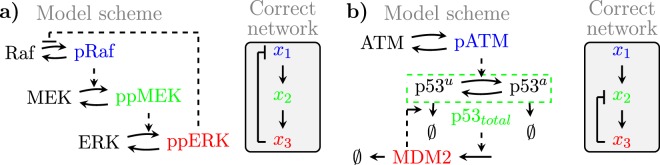
Two test-bed models. Shown are the reaction kinetic schemes (left) and the arising network structure (right) for **(a)** the MAPK system; **(b)** the p53 system. The coloured nodes (blue, green, red) indicate the measured species, which define the states of the models.

We used two test-bed examples to investigate noise propagation and impacts of the experimental design on the MRA estimates. The models are similar in that they both consist of three states (nodes) with two positive and one negative interactions (Fig. 2). However, both models feature very different equations, dynamics, and non-linear properties (Suppl. Fig. S1), allowing us to judge the generality of our results, and to investigate the impacts of moderate and strong non-linearities.

A model of signal transduction of the MAPK pathway upon EGF stimulation is illustrated in Fig. 2a. It consists of a three-tiered cascade of phosphorylation-dephosphorylation cycles in which pRaf phosphorylates and thereby activates MEK, which then activates ERK, which negatively feeds back to Raf^10^. Both MEK and ERK require phosphorylation at two sites to become fully active, which is for simplicity assumed to happen in a single reaction step for both proteins^11^. Variables *x*_1_, *x*_2_ and *x*_3_ represent protein activities. All reactions are modelled using Michaelis Menten type equations (Suppl. Fig. S1a). This system exhibits moderate non-linearity for the chosen parameters (Suppl. Fig. S1a).

The p53 model (Fig. 2b) is based on the core signalling system of the DNA damage response^12^. Here, ATM activates p53 by phosphorylation and protein stabilisation, p53 activates MDM2 by inducing gene expression, and MDM2 mediates a negative feedback loop to p53 by promoting p53 degradation^13^. In contrast to the MAPK system, the p53 system exhibits a strong degree of non-linearity (Suppl. Fig. S1b), including three ultra-sensitive Hill type equations for the reaction kinetics (Suppl. Fig. S1b).

### Problem formulation

MRA is a mathematical approach to reveal interaction strengths from steady-state perturbation data of a dynamic network of interacting modules. In our examples each module consists of a single protein, and we refer to them as nodes^5^. Considering the network at equilibrium, pairwise interaction strengths between nodes are characterized by quantifying the immediate change of the activity of one node of the network caused by a small change of another node, whereas the rest of the network is unaffected. Local Response Coefficients (LRCs) express these local effects among *N* nodes and are defined as the fractional change of the steady-state concentration of node *i* (${\bar{x}}_{i}$) with respect to that of node *j* (${\bar{x}}_{j}$), while keeping all other nodes ${\bar{x}}_{k}$, *k* ≠ *i*, *j*, at a constant level,

1 $${\rm{LRCs}}:\,{r}_{ij}^{true}=\frac{\partial \,\mathrm{ln}\,{\bar{x}}_{i}({\bar{x}}_{j},\,{\bar{x}}_{k})}{\partial \,\mathrm{ln}\,{\bar{x}}_{j}},\,{\bar{x}}_{k}={\rm{const}},\,k\ne i,\,j,\,i\ne j,\,i,\,j=\mathrm{1,}\,\ldots ,\,N.$$

These LRCs describe pairwise interactions between nodes when they act in isolation and are not directly accessible. A perturbation of one parameter *p*_*j*_, which specifically affects the activity of node *j*, spreads over the whole network, thus generating a global change of the equilibria of all nodes. This global change can be quantified from fold change measurements of concentrations relative to the unperturbed system. Formally, Global Response Coefficients (GRCs) are defined as the total derivative of the logarithm of the steady-state variables ($\mathrm{ln}\,{\bar{x}}_{i}$) with respect to the perturbed parameter (*p*_*j*_) (see exemplary network in Fig. 1a),

2 $${\rm{GRCs}}:\,{R}_{ij}^{true}=\frac{{\rm{d}}\,\mathrm{ln}\,{\bar{x}}_{i}({p}_{j})}{{\rm{d}}{p}_{j}}=\frac{1}{{\bar{x}}_{i}({p}_{j})}\frac{{\rm{d}}\,{\bar{x}}_{i}({p}_{j})}{{\rm{d}}\,{p}_{j}},\,i,\,j=\mathrm{1,}\,\ldots ,\,N\mathrm{.}$$

The corresponding MRA equations^5^

3 $$\sum _{j=\mathrm{1,}j\ne i}^{n}\,{r}_{ij}^{true}{R}_{jk}^{true}={R}_{ik}^{true}$$

establish a mathematically exact relationship between the GRCs and LRCs and can be used to extract LRCs from GRCs.

In our *in silico* study we assume that the investigated dynamical system can be described by a true underlying noise-free Ordinary Differential Equation (ODE) model $\dot{x}=f(x,\,p)$, with state variables $x=({x}_{1},\,\ldots ,\,{x}_{N})\in {{\mathbb{R}}}_{+}^{N}$ and parameters $p=({p}_{1},\,\ldots ,\,{p}_{N})\in {{\mathbb{R}}}_{+}^{N}$. The state variable *x*_*i*_ represents the activity of node *i*. The parameters *p*_*j*_ > 0, *j* = 1, …, *N*, are all equal to one in the nominal setting (control experiment), and can be varied to simulate the perturbation experiment affecting the corresponding node *j*. These parameters often affect preserved quantities such as total protein concentrations or production rates. The true LRCs ${r}_{ij}^{true},\,i\ne j$, are obtained by calculating the normalized entries of the Jacobian matrix at steady state, as described in^5^,

4 $${r}_{ij}^{true}={-(\frac{\partial {f}_{i}(x,p)}{\partial {x}_{j}}/\frac{\partial {f}_{i}(x,p)}{\partial {x}_{i}})\cdot (\frac{{x}_{j}}{{x}_{i}})|}_{ss},\,i\ne j,\,i,\,j=\mathrm{1,}\,\ldots ,\,N\mathrm{.}$$

When the underlying ODE system is not known, LRCs can be inferred from concentration measurements via two non-linear transformations. In a first transformation *T*_1_, differential GRCs are estimated from the steady states obtained in the control experiment $({\bar{x}}_{i}({p}_{j})=\,:{\bar{x}}_{i}^{0})$ and respective steady states in the perturbation experiments $({\bar{x}}_{i}({p}_{j}+{\rm{\Delta }}{p}_{j})=\,:{\bar{x}}_{i}^{j})$,

5 $${T}_{{1}}:\,{R}_{ij}^{\,true}{\rm{\Delta }}{p}_{j}\approx {\tilde{R}}_{ij}=\frac{{\bar{x}}_{i}^{j}-{\bar{x}}_{i}^{0}}{\frac{1}{2}({\bar{x}}_{i}^{j}+{\bar{x}}_{i}^{0})}=2\cdot \frac{{\bar{x}}_{i}^{j}-{\bar{x}}_{i}^{0}}{{\bar{x}}_{i}^{0}+{\bar{x}}_{i}^{j}},\,i,\,j=\mathrm{1,}\,\ldots ,\,N,$$

where we have approximated the derivative in (2) with finite differences and ${\bar{x}}_{i}^{0}$ with the average of ${\bar{x}}_{i}^{0}$ and ${\bar{x}}_{i}^{j}$. The *N* ⋅ (*N* − 1) LRCs are then obtained via substituting these ${\tilde{R}}_{ij}$ into equation (3), which corresponds to solving *N* linear systems with *N* − 1 equations in *N* − 1 independent variables each^5,14^,

6 $$\sum _{j=\mathrm{1,}j\ne i}^{n}\,{\tilde{r}}_{ij}{\tilde{R}}_{jk}={\tilde{R}}_{ik},\,k\ne i;\,i,\,k=\mathrm{1,}\,\ldots ,\,N\mathrm{.}$$

We note here that Δ*p*_*k*_ cancels out since it appears as a factor on both sides in this system. Due to the approximation (5), the values ${\tilde{r}}_{ij}$ obtained in this way are also an approximation of the true LRCs $({r}_{ij}^{true})$, and depend in particular on the perturbation strengths. In the following we will always consider ${\tilde{R}}_{ij}$ directly and thus refer to this measure simply as GRC. A second non-linear transformation, defined as *T*_2_, provides a solution for all coefficients ${\tilde{r}}_{ij}$. As shown in Suppl. S2 for *N* = 3, we can rewrite equation (6) as a linear system,

7 $${\bf{y}}({\tilde{R}}_{ij})=A({\tilde{R}}_{ij})\cdot {\bf{x}},$$

in which the vector **x** contains all unknowns ${\tilde{r}}_{ij}$, while ${\bf{y}}({\tilde{R}}_{ij})$ and *A*(${\tilde{R}}_{ij}$) are functions of the GRCs. The solution of this linear equation system, assuming that *A* has linearly independent columns, is given by

8 $${T}_{{2}}:\,{\bf{x}}={({A}^{T}A)}^{-1}{A}^{T}{\bf{y}}.$$

The variables ${\bar{x}}_{i}^{j},\,{\tilde{R}}_{ij}$ and ${\tilde{r}}_{ij}$ are assumed to be continuous functions of the perturbation parameters *p*_*j*_. For the following analysis, we refer to the difference

9 $${\rm{\Delta }}{r}_{ij}({p}_{j})=|{\tilde{r}}_{ij}({p}_{j})-{r}_{ij}^{true}|,\,i\ne j,\,i,\,j=\mathrm{1,}\,\ldots ,\,N$$

as intrinsic bias which results from the approximations (5) and (8).

Our two considered test-bed models for the MAPK and the p53 signalling pathways (see Suppl. S1) significantly differ in the courses of Δ*r*_*ij*_ over a large range of perturbation strengths *p*_*j*_ and thus can be considered as complementary examples concerning the approximation quality (8) and the validity of results in dependence of the perturbation strengths *p*_*j*_.

According to our statistical methodology, concentration measurements are described by random variables ${\bar{{\bf{z}}}}_{i}^{\mathrm{0,}j},\,i,\,j=\mathrm{1,}\,\ldots ,\,N$ (see Fig. 1a,b), whose distribution is a function of the noise-free steady-state values ${\bar{x}}_{i}^{\mathrm{0,}j}$. We consider a realistic error model consisting of a multiplicative and an independent additive part, similar to those suggested in^15^,

10 $${\bar{{\bf{z}}}}_{i}^{\mathrm{0,}j}={\bar{x}}_{i}^{\mathrm{0,}j}\cdot \eta +\varepsilon ,\,\eta  \sim \,\mathrm{log}\,{\mathscr{N}}\mathrm{(0,}\,{\sigma }_{\eta }^{2}),\,\varepsilon  \sim {\mathscr{N}}\mathrm{(0,}\,{\sigma }_{\varepsilon }^{2}),\,i,\,j=\mathrm{1,}\,\ldots ,\,N\mathrm{.}$$

The parameters *σ*_*η*_ and *σ*_*ε*_ denote the standard deviations of the proportional (log *η*) and additive (*ε*) measurement errors, respectively, ${\bar{x}}_{i}^{\mathrm{0,}j}$ are the simulated noise-free steady-state concentrations in the control (0) and perturbed (*j*) conditions, and ${\bar{{\bf{z}}}}_{i}^{\mathrm{0,}j}$ the resulting random variables representing the noisy simulated data.

We note here that due to the experimental procedure we often do not directly obtain concentrations but fold changes only. Thus, measurements ${\bar{z}}_{i}^{0}$ and ${\bar{z}}_{i}^{j}$ are realizations of random variables which are proportional to the real concentrations. As shown in Fig. 1b (left), we assume that measurements refer to signals detected via Western Blotting which have been normalized to a loading control. Without loss of generality, we neglect the proportionality factor specific for each blot. In fact, the GRCs calculated via equation (5) and hence the LRCs are independent of these factors, as long as the two samples ${\bar{z}}_{i}^{0}$ and ${\bar{z}}_{i}^{j}$ have been quantified in the same blot.

Due to the two non-linear transformations (5) and (8), the GRCs and LRCs are also random variables, which we express as **R**_*ij*_ and **r**_*ij*_ (see Fig. 1a,b). Given measurements of **R**_*ij*_, a solution of equation (7) is obtained by applying estimation methods. The simplest choice is to use Ordinary (Linear) Least Squares, whose solution has exactly the same form as equation (8). Changing the method corresponds to changing the operator *T*_2_, which remains a non-linear function of the GRCs in all cases.

Since it is impossible to derive the distributions of the LRCs directly from the error model (10) of the measurements, we applied a Monte Carlo approach in which we used our error model to simulate experimental data and propagated these to respective LRCs via the transformations *T*_1_ and *T*_2_.

Throughout the manuscript, bold letters indicate random variables and simple letters refer to realizations, as listed in Table 1, which collects the notation of all model quantities. To help the readers, we also collected a list of all abbreviations in Table 3 at the end of the Supplementary Material.

**Table 1:** Notation.

	True variable	Noise-free approximation	Random variable	Realization	Probability density function
Steady states	${\bar{x}}_{i}^{\mathrm{0,}j}$	—	${\bar{{\bf{z}}}}_{i}^{\mathrm{0,}j}$	${\bar{z}}_{i}^{\mathrm{0,}j}$	${p}_{{\bar{{\bf{z}}}}_{i}^{\mathrm{0,}j}}({\bar{z}}_{i}^{\mathrm{0,}j})$
GRCs	${R}_{ij}^{true}$	${\tilde{R}}_{ij}$	**R**_*ij*_	*R*_*ij*_	${p}_{{{\bf{R}}}_{ij}}({R}_{ij})$
LRCs	${r}_{ij}^{true}$	${\tilde{r}}_{ij}$	**r**_*ij*_	*r*_*ij*_	${p}_{{{\bf{r}}}_{ij}}({r}_{ij})$

### Solving the MRA equations results in heavy-tailed distributions for the estimated LRCs

We started our study by investigating the propagation of noise from the concentration measurements to the estimated LRCs as a basis to deduce strategies for an optimal design of experiments and estimation methods.

**Figure 3:**
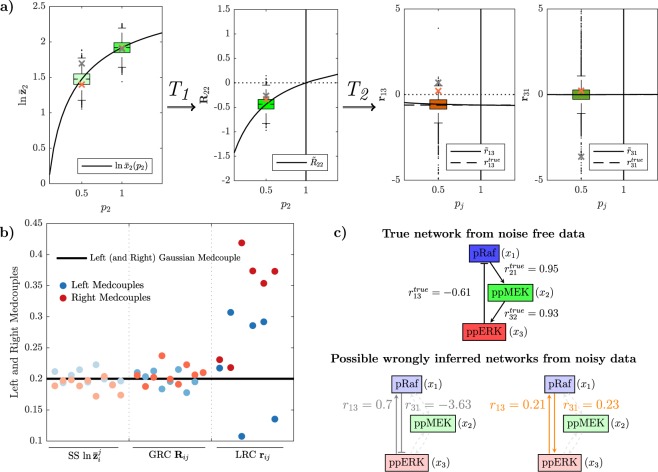
Non-linear propagation of measurement error in the MAPK test-bed model. (**a**) The curves represent the dependencies of steady states (left), GRCs (centre) and LRCs (right) over the changing parameters *p*_*j*_. Exemplary, on the left we show the distributions (boxplots) obtained from sampled noisy realizations of the steady state ${\bar{{\bf{z}}}}_{2}$ in the control experiment (*p*_2_ = 1) and in the 50% knockdown experiment of node 2 (*p*_2_ = 0.5). We generated *n* = 10,000 realizations via Monte Carlo simulations from the noise model (10) with parameters *σ*_*η*_ = 0.1 and *σ*_*ε*_ = 0.2. The ODE model with numerical values used for simulations is given in Suppl. S1. The variability in the measured steady states (left) translates into variability of the calculated GRCs (centre), which then translates into variability of the LRCs (right). Two sample points have been indicated by an orange and a grey cross and tracked during the transformations to illustrate consequences for network inference from individual samples. **(b)** Propagation of LMC and RMC values during the two-step transformation process reveals that heavy-tailedness is mainly introduced by the transformation *T*_2_. Numerical values are given in Suppl. Table S1. (**c**) True network structure of the MAPK test-bed model as obtained via equation (4). As a comparison, also the two realizations of the LRCs **r**_13_ and **r**_31_ that result from the two tracked orange and grey sample points are shown.

Therefore, we simulated the MAPK model with noise parameters that are in a biologically plausible range for Western blot data^16,17^. Exemplary results are shown in Fig. 3a, where resulting distributions are illustrated by box blots. While the variability of the resulting distributions of the GRCs is comparable to those of the inputs (Fig. 3a centre), we observe a much higher variability in the distributions of the LRCs (Fig. 3a right), which is mainly manifested in the number of outliers and the range covered by them. The complete set of distributions is given in the Supplementary material (Suppl. Figs S2–S4) and shows that these results are representative.

Driven by our analysis, we decided at that point to consider, besides standard measures for statistical dispersion such as interquartile ranges, also the amount of spread of the outliers, which is a measure for the degree of heavy-tailedness of an underlying distribution. Normalization of a signal obtained from a Western blot to a signal of a respective control experiment indeed corresponds to a transformation that may result in heavy-tailed distributions^18^. Since the tails of such distributions usually follow a power-law decay, the probability mass in the tails exceeds that of a Gaussian distribution, whose tails decay exponentially. As a consequence, samples from heavy-tailed distributions will contain more outliers which are spread over a larger range. A characteristic feature of heavy-tailed distributions is the fact that some or all moments do not exist. This severely impedes network reconstruction in our framework, since empirical estimators of moments are unstable due to the high occurrence of outliers. Empirical moments like the sample mean, the sample variance, or skewness and kurtosis, which are standard measures of asymmetry and tail-heaviness, do not provide meaningful estimates under these circumstances.

Thus, we decided to evaluate left and right medcouples (LMC and RMC)^19^ as suitable measures of left and right tail weights. The medcouple function (see Methods) was proposed as an efficient measure of the asymmetry of a univariate continuous distribution alternative to the classical skewness estimator^20^. The medcouple applied to one single side of the distribution leads to LMC and RMC, which are monotonically increasing functions of tail-heaviness. They are robust to outliers, since they only depend on quantiles and hence are suitable for heavy-tailed distributions. LMC and RMC values are put into context by comparison with the respective values for a standard Gaussian and Cauchy distribution, which are 0.2 and 0.5, respectively.

LMC and RMC values for concentration measurements, GRCs and LRCs are depicted in Fig. 3b. LMCs and RMCs for the distributions of the measurement data and of the GRCs are comparable to those of a Gaussian distribution. Respective values for the distributions of the estimated LRCs are considerably larger, indicating that heavy-tailedness is mainly introduced by the transformation *T*_2_. This increase might have severe consequences for network reconstruction, since it distorts estimation of moments of the LRCs such as the mean and the variance from samples. Evaluation of LMC and RMC values for the p53 test-bed model reveals similar results (Suppl. Fig. S5). Interestingly, MRA does not markedly affect the interquartile range (IQR) over the two transformations, which is a frequently used bulk-measure of variability (Suppl. Fig. S6).

We conclude that MRA amplifies the variability of the measurement noise in terms of degree of heavy-tailedness, while the IQR is not as much affected. Since heavy-tailedness is directly related to the occurrence of samples in the tails, which appear as outliers in the box plots, this impedes network reconstruction, as illustrated with two sample points indicated with orange and grey crosses and respective wrongly inferred network structures (Fig. 3c).

The question arises how we can optimize network reconstruction by influencing the distribution of the LRCs via experimental design and/or estimation procedures. In a first step we analyse how to best design the experiments regarding the choice of the perturbation strengths and the control strategy and subsequently investigate how to best handle multiple replicates.

### Large perturbations tend to improve the inference of pairwise node interactions

Since the GRCs and LRCs are defined as derivatives, a precise approximation via finite differences theoretically requires infinitesimal small perturbations, which is not feasible in practice. Moreover, noise deteriorates estimation of derivatives particularly from small differences. The question arises whether we are able to define perturbation strengths that constitute a good trade-off. For the MAPK test-bed model we observe that the noise-free approximated solution for the LRCs ${\tilde{r}}_{ij}$ is robust over a large range of perturbation parameters *p*_*j*_ and does not deviate much from the corresponding true value ${r}_{ij}^{true}$ (see left of Fig. 3a and Suppl. Fig. S4). This is different for the p53 test-bed model (Suppl. Fig. S9) and might also not be the case for other systems, which we usually don’t know a priori.

In order to answer our question, we compare the variability of the estimated LRCs resulting from different perturbation strengths. Therefore, we consider three knockdown experiments with downregulation of the 80%, 50% and 25% of the total protein concentrations with respect to the control experiment, and one overexpression experiment with 150% of total protein concentrations, resulting in a set of values for the perturbation strengths *p*_*j*_ ∈ {0.2, 0.5, 0.75, 1.5}.

**Figure 4:**
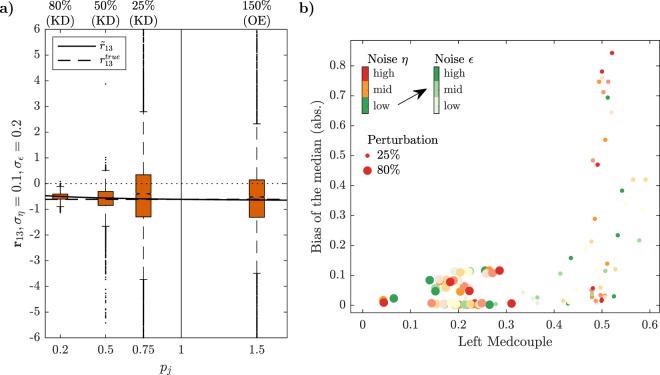
Effects of different perturbation strengths on network reconstruction. **(a)** Boxplots of the estimated LRC **r**_13_ of the MAPK test-bed model, describing the negative feedback from ERK to Raf, for different perturbation strengths: 80%, 50%, 25% knockdowns (KD) and 150% overexpression (OE) of the total protein concentrations. **(b)** Absolute values of the bias of the median versus LMC values for the entire set of of LRC values obtained with large (80%) or small (25%) knockdown strengths of the total protein concentrations. These statistics are given for different noise levels *σ*_*η*_ ∈ {0.05 (green), 0.1 (yellow), 0.2 (red)} and *σ*_*ε*_ ∈ {0.1, 0.2, 0.5} (indicated by increasing darkness).

As can be seen in Fig. 4a and Suppl. Fig. S10, the distributions of the estimated coefficients differ significantly in the four scenarios. The spread of the estimated coefficients is smallest for *p*_*j*_ = 0.2 and rapidly increases with decreasing perturbation strength, i.e. when *p*_*j*_ approaches one. The spread of the overexpression experiment is comparable to the 25% knockdown experiment, which is probably a result of the fact that we are in the saturated regime. We also observe a small and perturbation-dependent bias in the empirical estimate of the medians of all distributions.

To investigate the influence of the perturbation strength on accuracy and precision of the estimation more comprehensively, we collected values of the bias of the median and of the LMCs and RMCs for the 80% and the 25% knockdown experiments. Results are shown in Fig. 4b, where we have also visualized different noise levels *σ*_*η*_ and *σ*_*ε*_ with different colours and corresponding different shades. We observe a bias-spread trade-off between large and small perturbations. A low bias and a low LMC can only be obtained with large perturbations (large dots), while small perturbations lead to higher LMC values.

Increasing noise levels affect the bias markedly only for the small perturbation (small dots), which is true for all coefficients (Suppl. Fig. S11). The influence of increasing noise levels on LMC and RMC values is visible but moderate in the 80% knockdown experiment, while in case of 25% knockdown a marked effect can only be seen for very small multiplicative noise *σ*_*η*_ (Suppl. Fig. S12). Intriguingly, in most of the cases these quantities behave non-monotonically with respect to noise. Increasing noise does not necessarily imply larger bias or medcouples, which is probably due to the non-linear transformations *T*_1_ and *T*_2_. For the p53 test-bed model we observe similar trends (Suppl. Figs S13–S16), even though we observe a large bias of the median also for the large perturbation experiments here. From this analysis we conclude that larger perturbations are generally preferable, since they reduce the risk to infer erroneous network interactions.

### A simple control strategy is sufficient for the estimation of the LRCs

**Figure 5:**
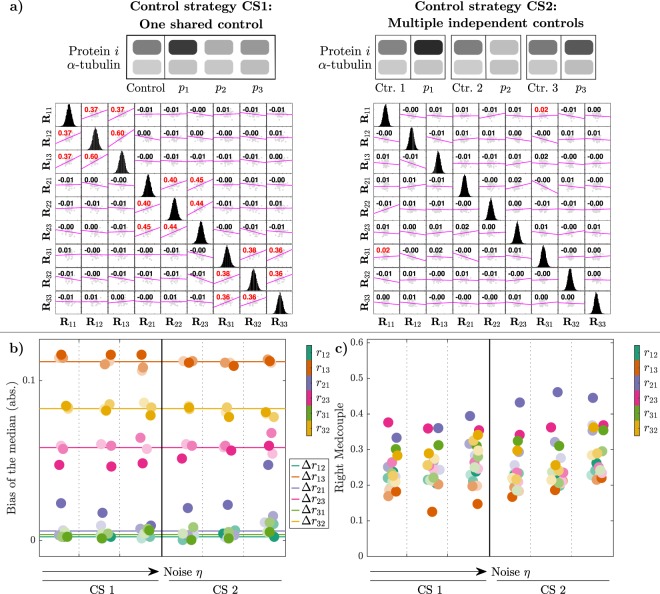
Effects of two alternative control strategies. **(a)** The first strategy (CS1) considers one single control realization for the calculation of all samples **R**_*ij*_ with *j* = 1, 2, 3, while the second strategy (CS2) performs independent control experiments for each perturbation experiment. Corresponding correlations can be seen in the **R**_*ij*_ scatter plot matrices. **(b)** Absolute values of the bias of the medians of the resulting LRCs **r**_*ij*_ for both control strategies CS1 (left) and CS1 (right) in dependence of different noise levels *σ*_*η*_ ∈ {0.05, 0.1, 0.2} and *σ*_*ε*_ ∈ {0.1, 0.2, 0.5}. For every value of *σ*_*η*_, i.e. for each specific column, the three shades of the LRCs correspond to the three (increasing) values of the standard deviation of the additive noise *σ*_*ε*_. Lines indicate intrinsic bias values for each LRC. **(c)** Same illustration for the RMC values.

The second component of the experiment design under investigation is the control strategy. Here we compare a single control for a node for all three perturbations (Fig. 5a left) versus individual controls for each perturbation (Fig. 5a right). The steady-state variable ${\bar{x}}_{i}^{0}$ of the control experiment appears in the GRCs ${\tilde{R}}_{ij}$ of all perturbation experiments *j* = 1, …, *N* (equation (5)). Simulating the first control strategy thus translates into using the same realization of the random variable ${\bar{{\bf{z}}}}_{i}^{0}$ to calculate the realizations *R*_*ij*_, *j* = 1, …, *N*, for fixed *i*, and results in block-wise positive correlations between the GRCs, as can be seen in the **R**_*ij*_ scatter plot matrix in Fig. 5a (left). These correlations disappear when performing multiple independent controls ${\bar{z}}_{i}^{0}$ for each perturbation experiment *j* (Fig. 5a right).

The choice of the control strategy determines the correlation among the coefficients **R**_*ij*_ with the same index *i*, but it is unclear whether it also has a marked effect on the LRCs **r**_*ij*_ and thus on network inference. In order to resolve this issue, we used the bias of the medians, the interquartile range (IQR), LMCs and RMCs as statistical measures of the distributions of the LRCs to compare the two control strategies. Since large perturbations have already turned out to be advantageous for MRA analysis, we simulated an 80% knockdown experiment and additionally also analysed the effect of increasing noise levels. As can be seen in Fig. 5b,c, we do not detect significant differences between the two control strategies with respect to the bias-spread trade-off. In fact, bias and RMCs behave similarly in the two cases. As before, we do not see a marked effect of increasing noise levels on the LRC distribution measures. These observations also generalize to the IQRs and the LMCs (Suppl. Fig. S18).

The horizontal lines shown in Fig. 5b represent the absolute values of the differences between the true LRCs and the LRCs resulting from the noise-free approximation, defined as Δ*r*_*ij*_ (equation (9)). For realistic noise levels, as used here, the bias of the medians is centred around this corresponding intrinsic bias, showing that the main contribution to the bias is caused by the approximation (5) rather than by the measurement noise. As before, there is no clear monotonic relation visible between the considered statistics and the levels of additive and multiplicative noise, respectively. The p53 test-bed model behaves very similar in this analysis (Suppl. Figs S19 and S20).

Taken together, since we could not observe marked differences of the LRC statistics between the two control strategies in both models, we advice experimenters to use the first control strategy of taking a single control measurement for a node for all corresponding perturbations, since this requires less samples.

### Using MRA with replicate mean values tends to outperform linear regression techniques

Generally, perturbation data contain several replicates of the same experiment. This raises the question of how to best handle these replicates during the MRA workflow. One solution is to calculate the mean over the replicates. Another, is the use of linear regression, for which several techniques have been suggested. The most common choice is to solve equation (7) by applying a least squares method, like Ordinary Least Squares (OLS) and Total Least Squares (TLS)^8^ (see Methods). But whether regression, and if so which, is better than using the mean over all replicates remains unclear.

Therefore we aim to solve the question about which estimation method, combined with the proper experimental design and data normalization, allows the best results in the terms of accuracy, precision and robustness of the LRCs estimates.

**Figure 6:**
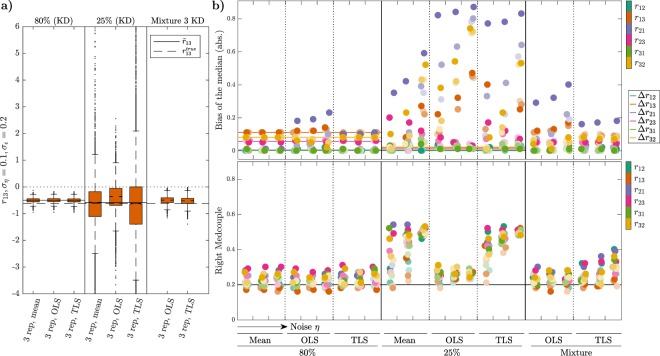
Effects of different estimation methods for the linear regression problem with multiple replicates. **(a)** Boxplots of the estimated LRC **r**_13_ of the MAPK model for two different perturbation strengths and different strategies to handle three replicates. First, the LRC are calculated by taking the mean values of the GRCs. Second, GRC replicates are taken individually into account and LRCs are obtained by solving OLS or TLS, respectively. The third column illustrates results from a mixture of measurements from three knockdown experiments with different perturbation strengths. **(b)** Absolute bias values of the estimated medians and RMC values for all LRCs and increasing levels of multiplicative and additive noise, *σ*_*η*_ ∈ {0.05, 0.1, 0.2} and *σ*_*ε*_ ∈ {0.1, 0.2, 0.5}. For every value of *σ*_*η*_, i.e. for a specific column, the three shades of the LRCs correspond to three (increasing) values of the standard deviation of the additive noise *σ*_*ε*_.

We compare results obtained with three replicates, which is the typically required number in many biological studies. In our simulations we mimic replicates by drawing independent realizations ${\bar{z}}_{i}^{\mathrm{0,}j}$, providing different realizations of the GRCs *R*_*ij*_. We considered the methods of taking the mean over the three obtained GRCs replicates and solving the linear regression problem (7); or determining GRC values for individual replicates and then applying either OLS or TLS from noisy values *R*_*ij*_, delivering one estimate of the LRCs *r*_*ij*_, *i*, *j* = 1, …, *N*. Moreover, we consider yet another experimental approach, in which we take multiple sample data not by repeating the same perturbation experiment but by varying the perturbation strengths *p*_*j*_, *j* = 1, 2, 3. Our choice is to mix three realizations obtained using three different knockdown strengths: 80%, 50% and 25% KD of the total protein concentrations. The results of our analysis are summarized in Fig. 6.

These results confirm that the experiment design with the large perturbation is superior compared to small perturbations or a combination of different perturbation strengths (see also Suppl. Figs S21 and S22). The considered measures for dispersion (LMC, RMC and IQR) are low and robust to noise for all three estimation approaches.

Interestingly, the mixture approach also delivers good results in terms of bias-spread trade-off (right part of Fig. 6, Suppl. Figs S21 and S22). As before, the OLS method results in a larger bias, but the dispersion measures are more sensitive to increasing noise if using TLS.

We can confirm that the experimental approach with the small perturbation strength delivers unsatisfactory results, leading to a high risk to reconstruct an erroneous network structure. Compared to the other two experimental designs, the bias is much larger and sensitive to noise with all three estimation methods: This holds especially true for the three non-zero coefficients *r*_21_, *r*_32_ and *r*_13_ (central part of Fig. 6b and Suppl. Fig. S22). The measures for dispersion are low for all coefficients only for very low noise and if using OLS.

Summarizing our results, we obtained the best estimation results in terms of accuracy, precision and robustness to noise by performing large perturbations and a simple control strategy. In terms of efficiency we recommend to use the simplest estimation method, which means to solve the regression problem (7) with the GRC means. In comparison, the mixture approach seems to be suboptimal in terms of bias-spread trade-off, but it might be beneficial for systems with higher non-linearities, as discussed in a later Subsection.

### Replicates increase precision, but not accuracy

The choice of the number of replicates is another important question for experimental design because of the trade-off between the experimental effort and cost, and the quality of the inferred results^21^. We address this issue by investigating how much the estimation of the LRCs is improved by increasing the number of replicates. We compare results obtained with one, three and six replicates. For each Monte Carlo run, we proceed by taking the mean value of these GRCs to further calculate one realization *r*_*ij*_ of the LRCs, which we have seen to be the most efficient estimation method, combined with large perturbations and the simple control strategy.

**Figure 7:**
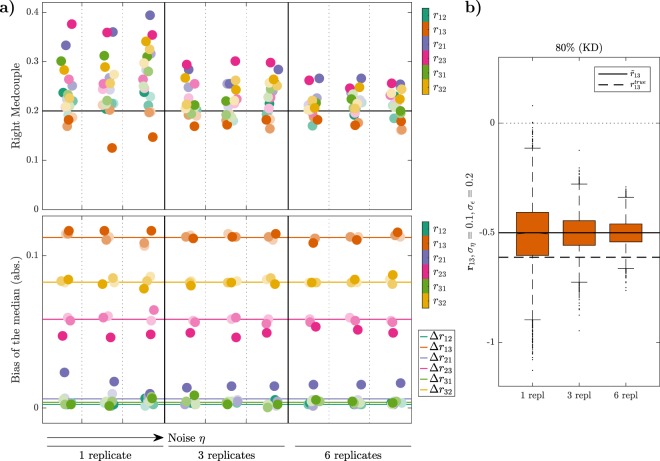
Effects of different numbers of replicates on network reconstruction. **(a)** RMCs and absolute bias values of the medians of the estimated distributions for the LRCs of the MAPK model for one, three and six replicates and different noise levels in the 80% KD perturbation experiments. Noise levels have been set to *σ*_*η*_ = {0.05, 0.1, 0.2} (different columns) and *σ*_*ε*_ = {0.1, 0.2, 0.5} (coded in different shades). The black line and the coloured lines indicate the RMC value for a normal distribution and the intrinsic bias values for each LRC, respectively. **(b)** Exemplary box plots of the LRC **r**_13_ for different numbers of replicates.

Results are depicted in Fig. 7. As expected, the precision of the estimation increases with the number of replicates, for both test-bed models. This manifests in a decrease of the considered measures for statistical dispersion, which are RMC and LMC values and the IQR, for all coefficients and noise levels and both test-bed models (Fig. 7a top, Suppl. Figs S26 and S28). In particular, RMC and LMC values converge to the value 0.2 of the standard Gaussian distribution. This effect is robust against increased multiplicative noise levels *η*.

In contrast, the biases in the medians are neither much affected by the number of replicates nor by the level of multiplicative noise, as can be seen in Fig. 7a (bottom) and Suppl. Fig. S28. In some cases increased additive noise *ε* (indicated by a darker shade of the coloured dots) leads to a larger bias, but not in a monotonic manner. As before, the medians rather coincide with the noise-free approximated values ${\tilde{r}}_{ij}$ (see also Suppl. Figs S25 and S27), whose deviations from the true values result from the choice of a large perturbation, showing that the bias in the medians is again dominated by the error of the approximation (5).

Summarizing, increasing the number of replicates reduces the dispersion of the distribution and therefore increases precision, but the bias cannot be eliminated, which restricts the accuracy of the estimates. We consider three replicates to be a good bias-spread trade-off, since all RMC values decrease below 0.3 when going from a single measurement to three replicates, while the decrease is much less pronounced when going from three to six replicates. Thus we recommend to use at least three replicates, and to include more depending on how much experimental effort is acceptable.

### Non-linearity induces bias, but large perturbations are still required for precision

In the MAPK model, the steady states show an approximately linear behaviour in dependence of the perturbation strengths in all cases (see Suppl. Fig. S1a), suggesting that the linear approximations (5) and (8) do not induce unduly large errors even for large perturbations. This was confirmed by our simulation results. When applying the MRA in practice, however, the course of the steady states of the system for varying perturbation strengths is not known, and it could also be highly non-linear. Do our recommendations and guidelines for an optimal performance of network reconstruction via MRA still hold true for such cases? In order to address this question, we used the p53 test-bed model as an example of a system whose steady states are non-linear functions of perturbation strengths (see Suppl. Fig. S1b). In this case the approximated LRCs ${\tilde{r}}_{ij}$ are sensitive to the choice of the perturbation strength and it is not clear a priori whether they are a good approximation of the true values ${r}_{ij}^{true}$ (compare Suppl. Figs S4 and S9).

We applied our MRA workflow to this test-bed model and performed the same analysis as before with the MAPK model. Summarizing, the results show that the most critical part is indeed the appearance of a large bias in the median of the distributions of the estimated **r**_*ij*_ if applying large perturbation experiments (see Suppl. Figs S23 (left parts) and S24a). This effect is related to the intrinsic bias Δ*r*_*ij*_(*p*_*j*_) and cannot be reduced by an increase in the number of replicates (see Suppl. Fig. S28a).

Nevertheless, the goal is to estimate the correct network structure, and therefore it is important to minimize the dispersion of the distributions of the estimated **r**_*ij*_. This holds especially if the intrinsic bias is significant for some of the LRCs, which is the case in the p53 example (Suppl. Fig. S23). In such cases it is necessary that the approximated LRCs ${\tilde{r}}_{ij}$ have the same sign as the true values, leading to qualitatively correctly estimated interactions. The trend of the spread of the estimated distributions shows that in general the lowest dispersion is still obtained with the largest perturbation experiment, in a similar way for all three computational approaches (see Suppl. Fig. S24b–d). In all these cases this behaviour is robust to increasing noise levels.

From these results we conclude that, due to the noise sensitivity, larger perturbations are generally still preferable, even for highly non-linear systems, since they reduce the risk to infer erroneous network interactions.

### Performance evaluation on the level of discrete network interactions corroborates our quantitative results

So far, we have investigated the influence of different experiment designs, estimation methods and noise levels on the statistical properties of the estimated LRCs. We have in particular focused on the bias of the median and on LMC and RMC values as measures for accuracy and precision of the individual estimates. In a final analysis step we transfer these results onto network inference, where the set of inferred LRCs is used to decide upon the network structure. The simplest way to do this is to arrange all LRCs according to their absolute value and to define a threshold for an interaction to be present or not. Sensitivity and specificity can then be calculated for an inferred network by a comparison with the true or a reference network. Doing this with varying threshold values, the Area Under the Curve (AUC) value is then an aggregated measure for the overall performance of the inference method independent of the threshold parameter. For such an analysis, it is not sufficient to look at each LRC separately. Here we applied an assessment method proposed in^9^, which is similar to a receiver-operator analysis, but also takes the signs of the inferred interactions into account. Depending on the percentage of correctly identified interactions, a normalized measure for the fit quality is assigned to an inferred network structure (see Fig. 1c), which is 0 in the worst and 1 in the best case. This overall measure for fit quality was determined for the different scenarios considered before and the distribution of this measure was investigated by sampling *n* = 10,000 network structures for each setting.

**Figure 8:**
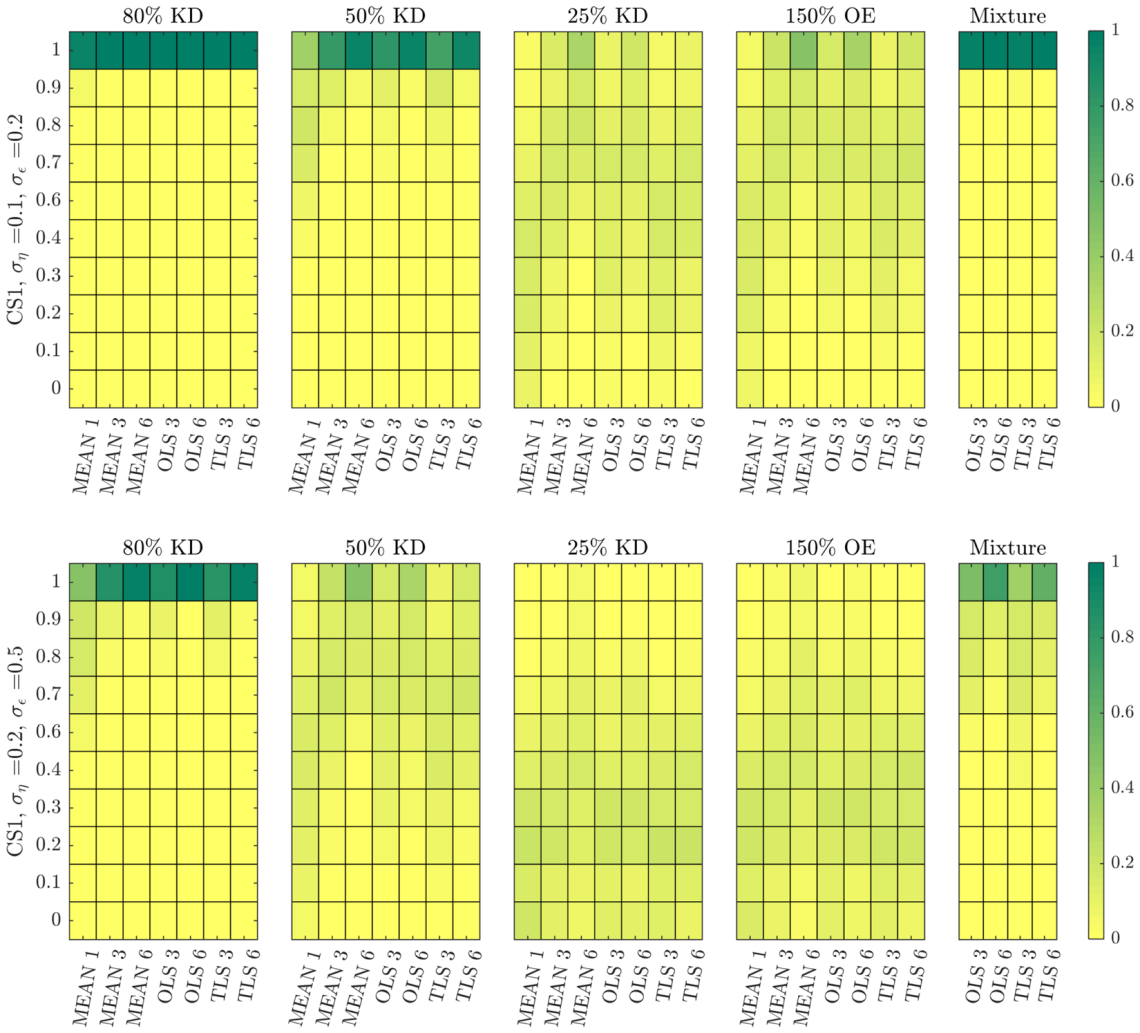
Performance evaluation of all MRA settings for network inference obtained with the MAPK test-bed model. Empirical distributions of fit quality of the inferred networks for different experimental designs and computational strategies for intermediate (top row) and high (bottom row) noise levels.

Results are shown in Fig. 8. Here, colour-coded empirical probability distributions of the discrete set of fit-quality values are shown for different settings. The first and second row depict results for intermediate and high noise levels, respectively. Different computational strategies and different perturbation strengths are compared. It can be seen that network inference works quite well in the 80% KD experiments for both noise levels and almost independent from the number of replicates and from the strategy to handle replicates. For intermediate noise levels, also the mixture method and the 50% KD perform very well, but are more sensitive to increasing noise levels. Both 25% KD and 150% OE perform worse in all scenarios. It can also be seen that results when considering replicates are not markedly different across almost all scenarios if averaging over the GRCs or when using OLS or TLS. We also compared these statistics across the two control strategies CS1 and CS2 (e.g. compare Suppl. Fig. S29a–d), which shows that the simple control strategy (CS1) is sufficient and there is no need to evaluate multiple independent control samples for each perturbation experiment. Similar results were obtained for the p53 model (Suppl. Fig. S30). Taken together, these results further confirm the conclusions drawn from the quantitative analyses in the previous sections: Firstly, due to noise, large perturbation are preferable, even for systems with a high degree of non-linearity. Secondly, it suffices to use a simple experimental strategy with one unperturbed control as reference for all perturbed conditions.

## Discussion

In this paper, we went through a comprehensive analysis of the effects of different experimental and estimation approaches for MRA on the goodness of network inference from noisy data, in terms of accuracy, precision and robustness. Our results led to some interesting findings. First, Monte Carlo simulations of concentration measurements with a realistic noise model for Western blot data clearly show a strong increase of heavy-tailedness, quantified in terms of LMC and RMC values, in the transformation from the GRCs to the LRCs, while respective values for concentration measurements and GRCs are quite similar (Fig. 3). This is a very relevant result, since heavy-tailedness deteriorates estimation of moments from samples, inducing as consequence a high risk of wrong outcomes for the network inference problem. In extreme cases, i.e. when certain moments are not defined, a stable estimation is not possible, even for large sample sizes. At least, this implies that concentrations and GRCs can be estimated more accurately from concentration measurements than LRCs. Second, for both test-bed models large perturbations are more favourable than smaller ones. Estimation of LRCs and hence network inference is much more accurate when using large perturbations. This is a non-trivial result, since estimation of the LRCs is done via a finite difference approximation of the GRCs in the MRA workflow, for which small differences would be beneficial in the absence of noise, since large differences imply an intrinsic bias. In particular, Fig. 4 shows a clear clustering of inferred LRCs according to the perturbation strengths: Results from 80% knockdown simulations show small biases and small LMC values, while 25% knockdowns show a much higher spread of bias values and consistently high LMC values. This leads to the clear advice to use large perturbations in the MRA workflow, even when the underlying model system features a considerable amount of non-linearity. Furthermore, regarding experiment design in the number of controls, our results indicate that a single control for different perturbation experiments, as often applied in practice, is sufficient (Fig. 5). While a single control causes correlations between GRCs, GRC marginals are not much affected. In particular, there is not much difference in the bias and RMC values of inferred LRC values among the two control strategies. The bias values are dominated by the intrinsic bias, and this is true for all tested noise levels. Regarding the required number of measurements and the estimation method, we advice to use the mean of at least three replicates. The spread of inferred LRCs decreases monotonically with the number of replicates, while the bias of the median is dominated by the intrinsic bias (Figs 6 and 7). Finally, our conclusions also hold true for the overall network inference problem, as evaluated in this study by a normalized quality measure for a classification problem (Fig. 8). Our results in particular show that learning the network topology is possible with very high accuracy also for high noise levels in our setting with the 80% knockdown experiments and few replicates.

As pointed out in the introduction, the effect of noise and variability in the data used for MRA network inference had already been an issue in earlier studies^1,8^. In a later work^22^, the authors developed an advanced version of MRA, combining it with a Bayesian model selection algorithm, relaxing also the restriction of required number of perturbation experiments to equal the number of nodes of the network. However, none of these are comparable in considering propagation of noise from concentration measurements via GRCs and LRCs to network topology inference in a consistent stochastic framework with realistic noise assumptions. These studies also use Monte Carlo techniques, but start with i.i.d. normal distributions directly on the GRC values, and also completely neglect the effects of heavy-tailedness. They are also lacking concrete recommendations for experimental design and computational methodology regarding MRA based network inference.

As with all inference methods, our methodology has some limitations. The MRA framework itself assumes a continuous functional dependence between perturbation parameters and steady states of the system. This excludes for instance systems which exhibit bifurcations, as they appear for example in positive feedback systems which exhibit multi-stability. For those systems, the theory only holds as long as the perturbation does not induce a switch of the system to a different fixed point branch. It might be difficult to decide whether this is indeed the case in real settings, where the underlying dynamical system is not known. Furthermore, there might be potential for improvement regarding methodology to solve the regression problem to calculate the LRCs from the GRCs. Methods like feasible generalized least squares or Maximum Likelihood estimation might be beneficial in this respect. Finally, evaluation of our findings and recommendations in a setting with real experimental data is an open issue for the future.

## Methods

### Medcouple

Given a set of *n* independent samples {*x*_1_, ..., *x*_*n*_} from a continuous univariate distribution, with median *m*_*n*_, the medcouple is defined as

$${\rm{MC}}=\mathop{{\rm{med}}}\limits_{{x}_{i}\le {m}_{n}\le {x}_{j}}h({x}_{i},\,{x}_{j}),\,{\rm{with}}\,h({x}_{i},\,{x}_{j})=\frac{({x}_{j}-{m}_{n})-({m}_{n}-{x}_{i})}{{x}_{j}-{x}_{i}},\,\forall {x}_{j}\ne {x}_{i}.$$

The kernel function *h*(*x*_*i*_, *x*_*j*_) measures the (normalized) difference between the distances of *x*_*i*_ and *x*_*j*_ to the median. The medcouple represents a robust measure of the asymmetry of a distribution, which can be computed also for distributions without finite moments, which is not the case for the classical skewness coefficient^20^. As robust measure of tail weight, the authors propose to apply the medcouple only to one single side of the distribution, leading to Left Medcouple (LMC) and Right Medcouple (RMC)^19^:

$${\rm{LMC}}=-\,{\rm{MC}}(x < {m}_{n})\,{\rm{and}}\,{\rm{RMC}}={\rm{MC}}(x > {m}_{n}\mathrm{).}$$

The calculation of such quantities for all datasets in our study was performed with the MATLAB toolbox LIBRA^23^, developed by the same authors, which can be downloaded from https://wis.kuleuven.be/stat/robust/LIBRA/LIBRA-home.

### MRA estimation methods

For the estimation problem, we have to solve equation (7), **y** = *A*⋅**x**, which is a linear regression model, in the unknown variable **x**. Assuming no error in the regression variables, i.e. in the entries of the matrix *A*, and i.i.d. normal errors in the variable **y**, we obtain the well known ordinary least squares (OLS) solution, given in equation (8). However, this assumption is wrong, since the entries in the matrix *A* are also affected by noise, being samples of GRCs. One option is to consider error-in-variables models, such as total least squares (TLS), whose computation requires singular value decomposition and is presented in^8^.

## Electronic supplementary material

Supplementary material

## Data Availability

The datasets generated during and/or analysed in this study, as well as the Matlab code and functions and the SBML versions of the models, can be downloaded from https://zenodo.org/record/1434792#.W6pBmC-B2MI; or using the 10.5281/zenodo.1434792. The MAPK and p53 models are available in standard SBML format from the EMBL-EBI BioModels repository under accession numbers MODEL1809260001 and MODEL1809260002, respectively.
