# Supplementary material for: Impact of measurement noise, experimental design, and estimation methods on Modular Response Analysis based network reconstruction
Source: Sci Rep. 2018 Nov 1;8:16217. doi: 10.1038/s41598-018-34353-3 (PMC6212399; doi:10.1038/s41598-018-34353-3)
Supplement: Supplementary file 1 — Supplementary material [file 41598_2018_34353_MOESM1_ESM.pdf]

Impact of measurement noise, experimental design, and estimation methods on Modular Response Analysis based network reconstruction - SUPPLEMENTARY MATERIAL

Caterina Thomaseth[1,+], Dirk Fey[2,+], Tapesh Santra[2], Oleksii Rukhlenko[2], Nicole E. Radde[1,*,#], and Boris Kholodenko[2,#]

[1]University of Stuttgart, Institute for Systems Theory and Automatic Control, Stuttgart, Germany
[2]University College Dublin, Systems Biology Ireland, Dublin, Ireland
[+]Equal contributors
[#]Joint last authors
[*]nicole.radde@ist.uni-stuttgart.de

Contents

S1 - ODE test-bed models 1
S2 - Linear regression model for the LRCs 3
S3 - Variation of the steady-state variables, GRCs and LRCs for changing perturbation strengths 4
S4 - Experiment design: choice of the perturbation strength 12
S5 - Experiment design: choice of the control strategy 18
S6 - Estimation methods to handle multiple replicates and solve the linear regression problem 22
S7 - Experiment design: number of replicates 27
S8 - Performance evaluation 31

S1 - ODE test-bed models

Suppl. Fig. S1 shows the two test-bed models which are used in this study. Both models are based on previously published models for the MAPK [REFKholodenko2006] and p53 [REFFey2016] systems. Parameters have been chosen in order to obtain an EGF-induced MAPK model that behaves relatively linear and a p53 DNA-damage response model that behaves strongly nonlinear. This reflects the observed behavior of these systems [REFKholodenko2010], [REFPurvis2012].

A model of signal transduction of the MAPK pathway upon EGF stimulation is illustrated in Suppl. Fig. S1a. It consists of a three-tiered cascade of phosphorylation-dephosphorylation cycles in which pRaf phosphorylates and thereby activates MEK, which then activates ERK, which negatively feeds back to Raf. Both MEK and ERK require phosphorylation at two sites to become fully activated, which is for simplicity assumed to happen in a single reaction step for both proteins. This system is described by a dynamical model in the form of Ordinary Differential Equations $\dot{x} = f(x,\theta)$, $x \in \mathbb{R}^3_+$. The state variables $x(t) = [x_1(t), x_2(t), x_3(t)]^\top$ refer to the active states of the three proteins pRaf, ppMEK and ppERK. The input $u(t) = \text{EGF}$ is set to a constant value $u(t) = 1$. The initial conditions of the state variables are set to zero, i.e. there are no active states at time $t = 0$. The total concentrations of the three states are $\text{Raf}_{\text{TOT}}, \text{MEK}_{\text{TOT}}, \text{ERK}_{\text{TOT}} = 20$. The kinetic rates and other parameter values are: $k_1 = 5, K^+_{m1} = 20, V_{m1} = 10, K^-_{m1} = 20, k_2 = 3, K^+_{m2} = 20, V_{m2} = 10, K^-_{m2} = 20, k_3 = 1, K^+_{m3} = 20, V_{m3} = 10, K^-_{m3} = 20, K_{mf} = 5$. Perturbation parameter p_1, p_2 and p_3 correspond to fold changes in total protein amounts. One after the other they are set to some defined value smaller or greater than 1, the same for all perturbation experiments. To obtain the simulation results presented in the manuscript, we considered the values $p_j \in \{0.2, 0.5, 0.75, 1.5\}, \forall j = 1,2,3$, that represent 80%, 50% and 25% knockdown or 50% overexpression of the total protein concentrations.

Suppl. Fig. S1b illustrates activation of p53 by pATM. Active p53 triggers expression of MDM2, which is in turn involved in the degradation of p53, resulting in a negative feedback loop. Both p53 and MDM2 are subject to synthesis and degradation, and model variables x_1, x_2 and x_3 correspond to pATM, p53 total amount and MDM2, respectively. As before, perturbation

parameters p_1, p_2 and p_3 describe fold changes in ATM total amount, and in p53 and MDM2 synthesis rates.
The initial conditions of the state variables are set to zero, i.e. there are no active states at time $t = 0$. The input is set to a constant value $u(t) = 1$. The kinetic rates and other parameter values are: $k_1 = 3, K_1 = 0.5, k_2 = 5, K2 = 0.5, k_3 = 1, n_5 = 5, K_5 = 0.1, k_4 = 1, KD = 0.01, k_6 = 1, n_6 = 5, K_6 = 0.5, k_7 = 1$. The total concentration of ATM is $\mathrm{ATM_{TOT}} = 1$.

In both subfigures, graphs indicate the steady states of the system variables as functions of the perturbation parameters. While these curves can well be approximated by linear functions in the first case, they show a more pronounced non-linear behaviour in the second case.

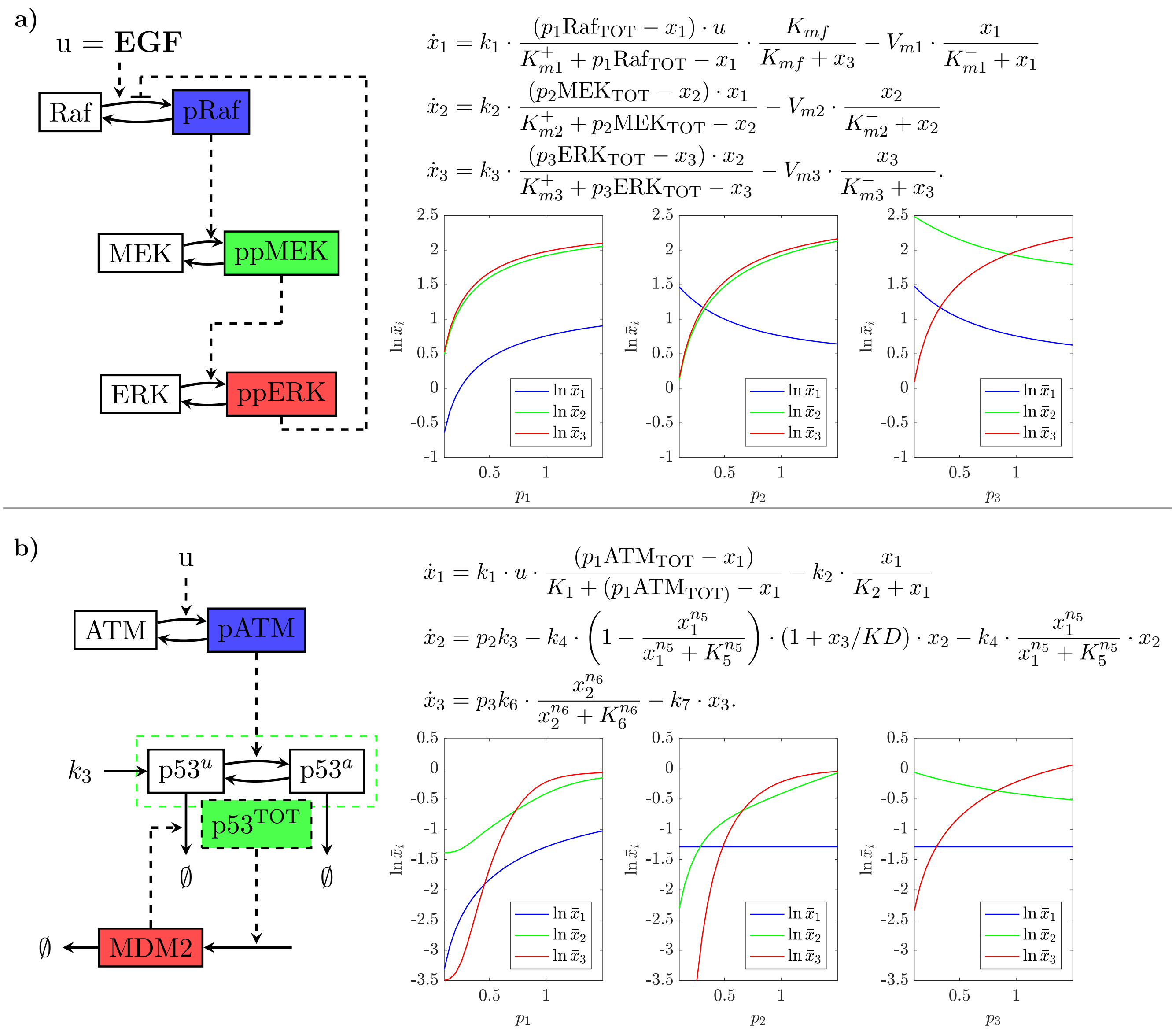

Figure S1. Two test-bed models. Shown are the reaction kinetic schemes (left), the ODE system (right top) and the dependencies of the logarithm of the state variables on the perturbation parameters (right bottom) for **(a)** the MAPK system; **(b)** the p53 system.

S2 - Linear regression model for the LRCs

For $N = 3$ we can rewrite equation (5) of the manuscript as the following set of 3 linear systems:

$$
\begin{aligned}
(i=1): \quad & \tilde{r}_{12}\tilde{R}_{22} + \tilde{r}_{13}\tilde{R}_{32} = \tilde{R}_{12} \\
& \tilde{r}_{12}\tilde{R}_{23} + \tilde{r}_{13}\tilde{R}_{33} = \tilde{R}_{13} \\
(i=2): \quad & \tilde{r}_{21}\tilde{R}_{11} + \tilde{r}_{23}\tilde{R}_{31} = \tilde{R}_{21} \\
& \tilde{r}_{21}\tilde{R}_{13} + \tilde{r}_{23}\tilde{R}_{33} = \tilde{R}_{23} \\
(i=3): \quad & \tilde{r}_{31}\tilde{R}_{11} + \tilde{r}_{32}\tilde{R}_{21} = \tilde{R}_{31} \\
& \tilde{r}_{31}\tilde{R}_{12} + \tilde{r}_{32}\tilde{R}_{22} = \tilde{R}_{32}
\end{aligned}
$$

Each of the three systems with two equations in two independent variables can be rewritten in the following matrix form:

$$
\begin{aligned}
(i=1): \begin{bmatrix} \tilde{R}_{12} \\ \tilde{R}_{13} \end{bmatrix} &= \begin{bmatrix} \tilde{R}_{22} & \tilde{R}_{32} \\ \tilde{R}_{23} & \tilde{R}_{33} \end{bmatrix} \begin{bmatrix} \tilde{r}_{12} \\ \tilde{r}_{13} \end{bmatrix} \\
(i=2): \begin{bmatrix} \tilde{R}_{21} \\ \tilde{R}_{23} \end{bmatrix} &= \begin{bmatrix} \tilde{R}_{11} & \tilde{R}_{31} \\ \tilde{R}_{13} & \tilde{R}_{33} \end{bmatrix} \begin{bmatrix} \tilde{r}_{21} \\ \tilde{r}_{23} \end{bmatrix} \\
(i=3): \begin{bmatrix} \tilde{R}_{31} \\ \tilde{R}_{32} \end{bmatrix} &= \begin{bmatrix} \tilde{R}_{11} & \tilde{R}_{21} \\ \tilde{R}_{12} & \tilde{R}_{22} \end{bmatrix} \begin{bmatrix} \tilde{r}_{31} \\ \tilde{r}_{32} \end{bmatrix} .
\end{aligned}
$$

By stacking all unknowns $\tilde{r}_{ij}, i, j = 1,2,3$, in one single vector $\mathbf{x}$, we obtain the following 6 dimensional linear system $\mathbf{y} = A \cdot \mathbf{x}$:

$$
\begin{bmatrix} \tilde{R}_{12} \\ \tilde{R}_{13} \\ \tilde{R}_{21} \\ \tilde{R}_{23} \\ \tilde{R}_{31} \\ \tilde{R}_{32} \end{bmatrix} = \begin{bmatrix} \tilde{R}_{22} & \tilde{R}_{32} & 0 & 0 & 0 & 0 \\ \tilde{R}_{23} & \tilde{R}_{33} & 0 & 0 & 0 & 0 \\ 0 & 0 & \tilde{R}_{11} & \tilde{R}_{31} & 0 & 0 \\ 0 & 0 & \tilde{R}_{13} & \tilde{R}_{33} & 0 & 0 \\ 0 & 0 & 0 & 0 & \tilde{R}_{11} & \tilde{R}_{21} \\ 0 & 0 & 0 & 0 & \tilde{R}_{12} & \tilde{R}_{22} \end{bmatrix} \begin{bmatrix} \tilde{r}_{12} \\ \tilde{r}_{13} \\ \tilde{r}_{21} \\ \tilde{r}_{23} \\ \tilde{r}_{31} \\ \tilde{r}_{32} \end{bmatrix} .
$$

S3 - Variation of the steady-state variables, GRCs and LRCs for changing perturbation strengths

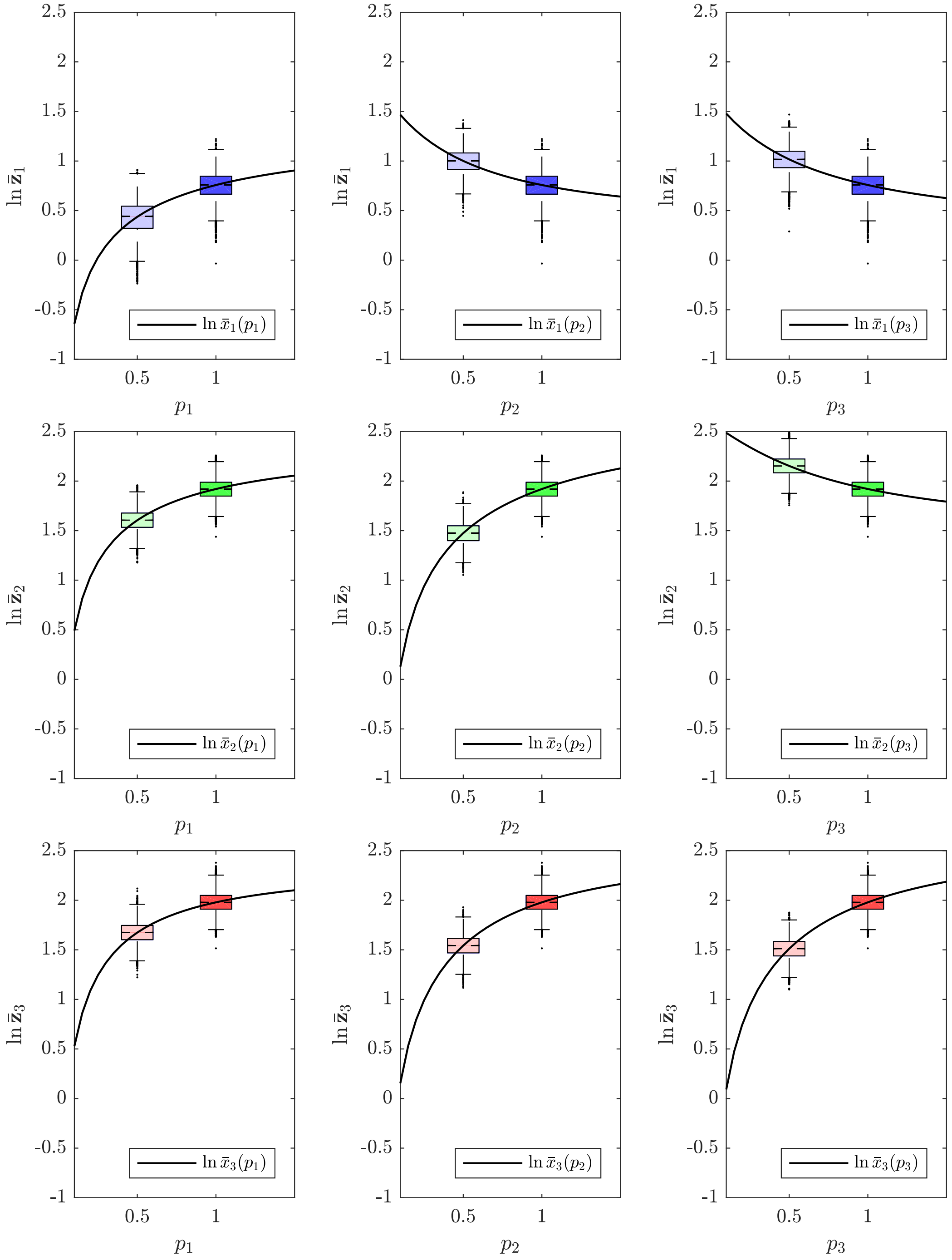

Figure S2. Nonlinear variation of the steady-state variables for changing perturbation strengths for the MAPK test-bed model. The variability of the measured steady states $\bar{\mathbf{z}}_i, i = 1,2,3$ is shown in the control experiment ($p_j = 1, j = 1,2,3$) and in the 50% knockdown experiments ($p_j = 0.5, j = 1,2,3$). We generated $n = 10,000$ realizations via Monte Carlo simulations from the noise model (9) with parameters $\sigma_\eta = 0.1$ and $\sigma_\varepsilon = 0.2$. The continuous solid lines correspond to the noise free trend of the logarithm of the steady states for changing perturbation parameters ($\ln \bar{x}_i(p_j)$).

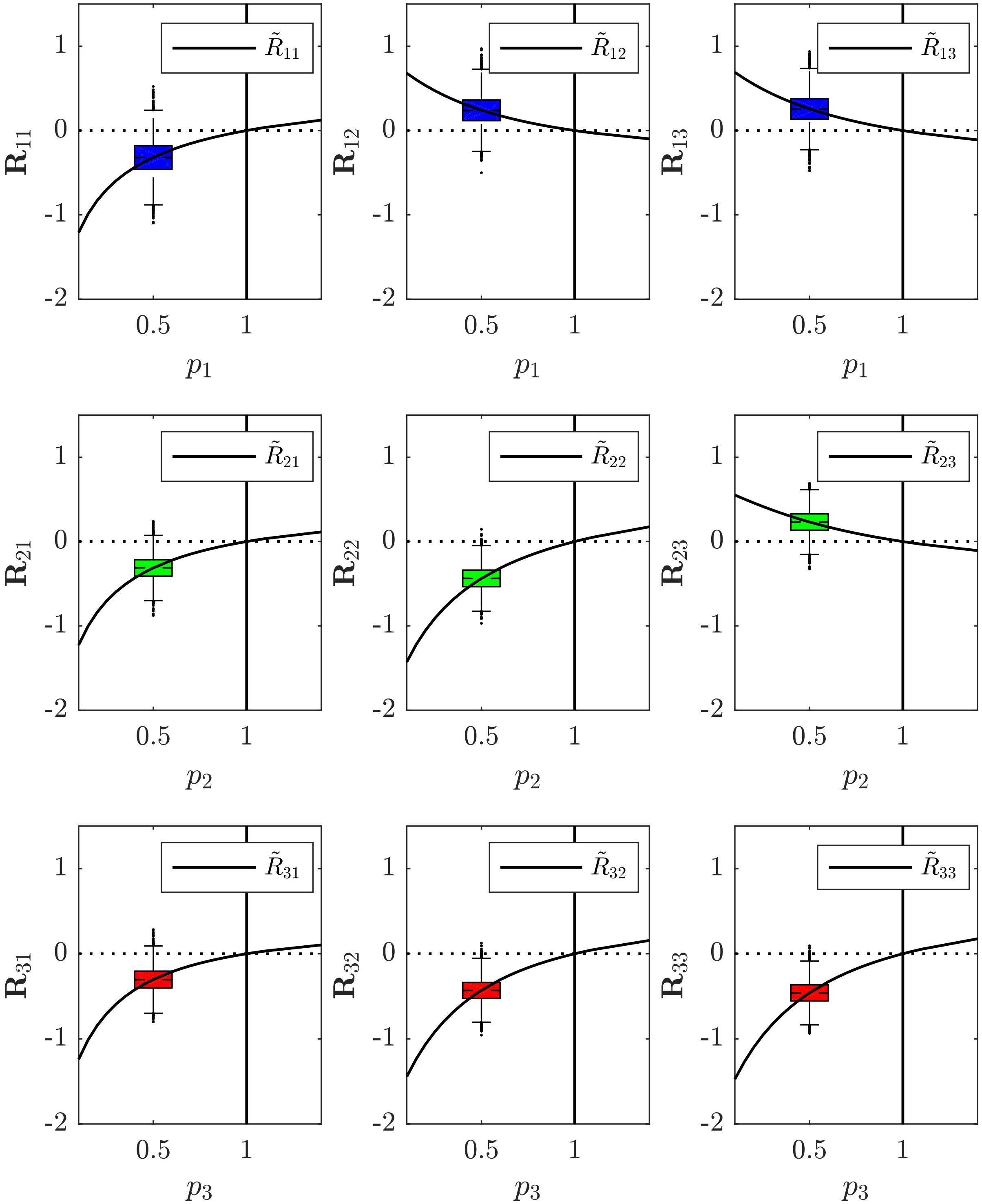

Figure S3. Nonlinear variation of the GRCs for changing perturbation strengths for the MAPK test-bed model. The variability of the GRCs $\mathbf{R}_{ij}, i, j = 1, 2, 3$ is obtained from the sampled steady-state realizations from Fig. S2 in the case of 50% knockdown experiments ($p_j = 0.5, j = 1, 2, 3$). We generated $n = 10,000$ realizations via Monte Carlo simulations from the noise model (9) with parameters $\sigma_\eta = 0.1$ and $\sigma_\varepsilon = 0.2$. The continuous solid lines correspond to the noise free trend of the approximation of the GRCs for changing perturbation parameters ($\tilde{R}_{ij}(p_j)$).

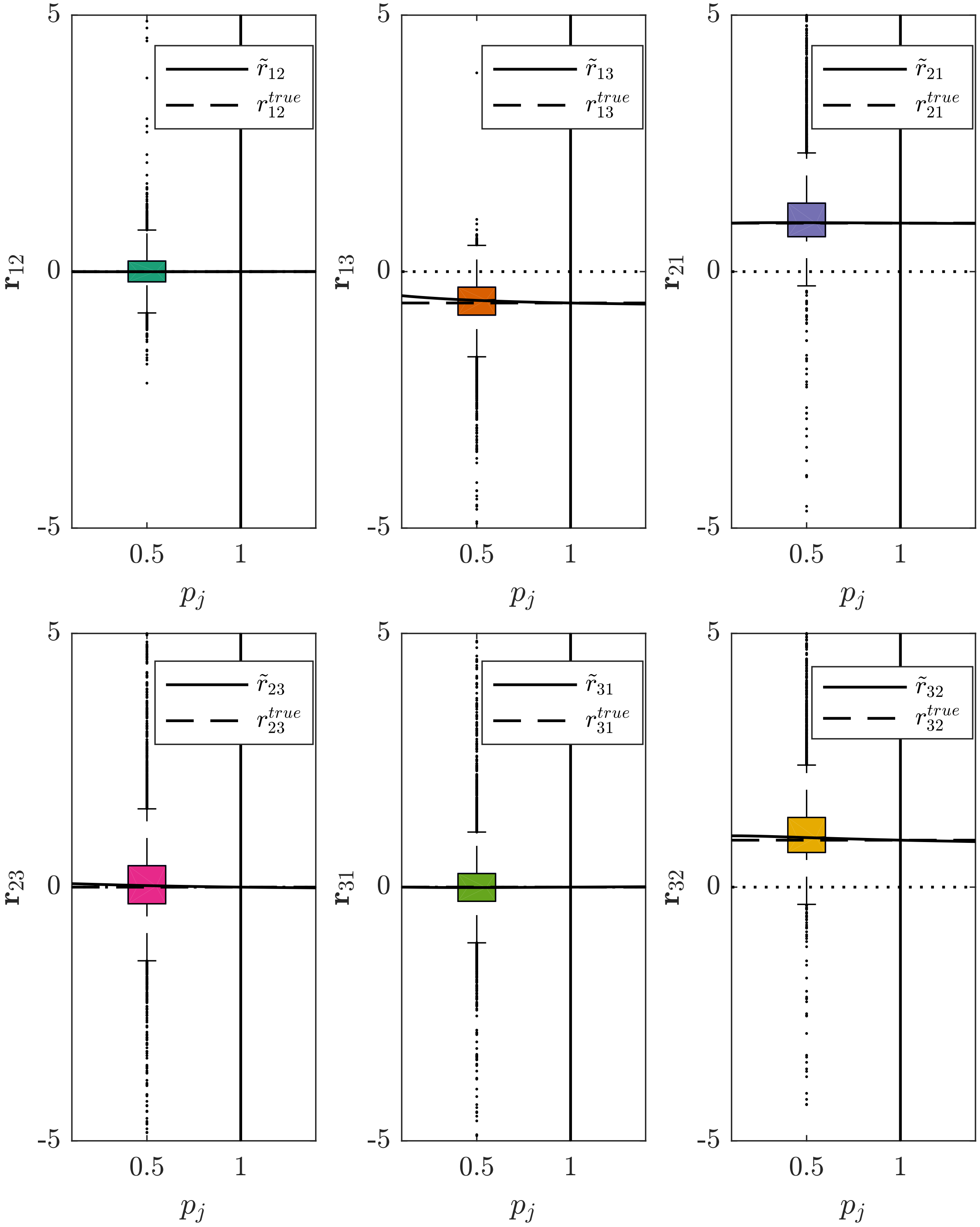

Figure S4. Nonlinear variation of the LRCs for changing perturbation strengths for the MAPK test-bed model. The variability of the LRCs $\mathbf{r}_{ij}, i, j = 1,2,3$ is obtained from the sampled steady-state realizations from Fig. S2 in the case of 50% knockdown experiments ($p_j = 0.5, j = 1,2,3$). We generated $n = 10{,}000$ realizations via Monte Carlo simulations from the noise model (9) with parameters $\sigma_\eta = 0.1$ and $\sigma_\varepsilon = 0.2$. The continuous solid lines correspond to the noise free trend of the approximation of the LRCs for changing perturbation parameters ($\tilde{r}_{ij}(p_j)$).

Table S1. **(a)** Left and **(b)** right medcouples of steady states, GRCs and LRCs for the MAPK test-bed model

(a) Left medcouples

SS $\ln \bar{\mathbf{z}}_i^j$		GRC $\mathbf{R}_{ij}$		LRC $\mathbf{r}_{ij}$	
$\ln \bar{\mathbf{x}}_1^1$	0.2118	$\mathbf{R}_{11}$	0.2103	$\mathbf{r}_{11}$	—
$\ln \bar{\mathbf{x}}_1^2$	0.1937	$\mathbf{R}_{12}$	0.2037	$\mathbf{r}_{12}$	0.2173
$\ln \bar{\mathbf{x}}_1^3$	0.2060	$\mathbf{R}_{13}$	0.2127	$\mathbf{r}_{13}$	0.3070
$\ln \bar{\mathbf{x}}_2^1$	0.2148	$\mathbf{R}_{21}$	0.1838	$\mathbf{r}_{21}$	0.1076
$\ln \bar{\mathbf{x}}_2^2$	0.1948	$\mathbf{R}_{22}$	0.1958	$\mathbf{r}_{22}$	—
$\ln \bar{\mathbf{x}}_2^3$	0.2228	$\mathbf{R}_{23}$	0.2151	$\mathbf{r}_{23}$	0.2857
$\ln \bar{\mathbf{x}}_3^1$	0.1997	$\mathbf{R}_{31}$	0.1779	$\mathbf{r}_{31}$	0.2918
$\ln \bar{\mathbf{x}}_3^2$	0.1909	$\mathbf{R}_{32}$	0.1953	$\mathbf{r}_{32}$	0.1353
$\ln \bar{\mathbf{x}}_3^3$	0.1966	$\mathbf{R}_{33}$	0.2099	$\mathbf{r}_{33}$	—

(b) Right medcouples

SS $\ln \bar{\mathbf{z}}_i^j$		GRC $\mathbf{R}_{ij}$		LRC $\mathbf{r}_{ij}$	
$\ln \bar{\mathbf{x}}_1^1$	0.1888	$\mathbf{R}_{11}$	0.2057	$\mathbf{r}_{11}$	—
$\ln \bar{\mathbf{x}}_1^2$	0.1974	$\mathbf{R}_{12}$	0.1878	$\mathbf{r}_{12}$	0.2309
$\ln \bar{\mathbf{x}}_1^3$	0.1985	$\mathbf{R}_{13}$	0.2026	$\mathbf{r}_{13}$	0.2182
$\ln \bar{\mathbf{x}}_2^1$	0.1887	$\mathbf{R}_{21}$	0.2372	$\mathbf{r}_{21}$	0.4188
$\ln \bar{\mathbf{x}}_2^2$	0.1976	$\mathbf{R}_{22}$	0.1996	$\mathbf{r}_{22}$	—
$\ln \bar{\mathbf{x}}_2^3$	0.1721	$\mathbf{R}_{23}$	0.1914	$\mathbf{r}_{23}$	0.3737
$\ln \bar{\mathbf{x}}_3^1$	0.2041	$\mathbf{R}_{31}$	0.2227	$\mathbf{r}_{31}$	0.3537
$\ln \bar{\mathbf{x}}_3^2$	0.1901	$\mathbf{R}_{32}$	0.2065	$\mathbf{r}_{32}$	0.3731
$\ln \bar{\mathbf{x}}_3^3$	0.1739	$\mathbf{R}_{33}$	0.2102	$\mathbf{r}_{33}$	—

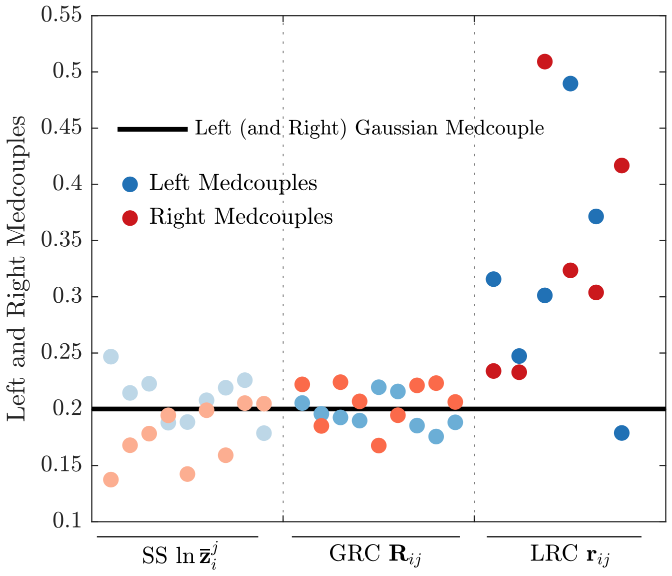

Figure S5. Left and right medcouples for the p53 test-bed model. Propagation of LMC and RMC values during the two-step transformation process reveals that heavy-tailedness is mainly introduced by the transformation T_2. Numerical values are given in Suppl. Table S2.

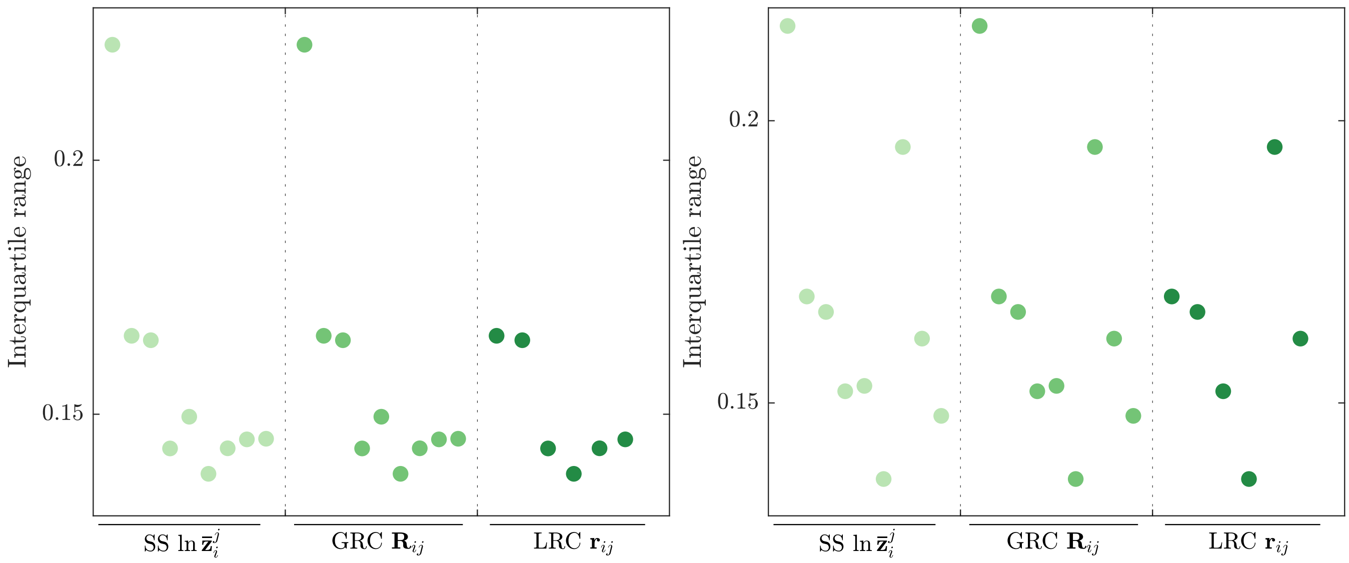

(a) MAPK

(b) p53

Figure S6. Interquartile ranges for both test-bed models. **(a)** MAPK test-bed model; **(b)** p53 test-bed model.

Table S2. **(a)** Left and **(b)** right medcouples of steady states, GRCs and LRCs for the p53 test-bed model

(a) Left medcouples

SS $\ln \bar{\mathbf{z}}_i^j$		GRC $\mathbf{R}_{ij}$		LRC $\mathbf{r}_{ij}$	
$\ln \bar{\mathbf{x}}_1^1$	0.2468	$\mathbf{R}_{11}$	0.2056	$\mathbf{r}_{11}$	—
$\ln \bar{\mathbf{x}}_1^2$	0.2146	$\mathbf{R}_{12}$	0.1959	$\mathbf{r}_{12}$	0.3157
$\ln \bar{\mathbf{x}}_1^3$	0.2227	$\mathbf{R}_{13}$	0.1927	$\mathbf{r}_{13}$	0.2473
$\ln \bar{\mathbf{x}}_2^1$	0.1881	$\mathbf{R}_{21}$	0.1899	$\mathbf{r}_{21}$	0.3013
$\ln \bar{\mathbf{x}}_2^2$	0.1886	$\mathbf{R}_{22}$	0.2196	$\mathbf{r}_{22}$	—
$\ln \bar{\mathbf{x}}_2^3$	0.2080	$\mathbf{R}_{23}$	0.2158	$\mathbf{r}_{23}$	0.4896
$\ln \bar{\mathbf{x}}_3^1$	0.2192	$\mathbf{R}_{31}$	0.1855	$\mathbf{r}_{31}$	0.3715
$\ln \bar{\mathbf{x}}_3^2$	0.2259	$\mathbf{R}_{32}$	0.1757	$\mathbf{r}_{32}$	0.1789
$\ln \bar{\mathbf{x}}_3^3$	0.1788	$\mathbf{R}_{33}$	0.1884	$\mathbf{r}_{33}$	—

(b) Right medcouples

SS $\ln \bar{\mathbf{z}}_i^j$		GRC $\mathbf{R}_{ij}$		LRC $\mathbf{r}_{ij}$	
$\ln \bar{\mathbf{x}}_1^1$	0.1374	$\mathbf{R}_{11}$	0.2221	$\mathbf{r}_{11}$	—
$\ln \bar{\mathbf{x}}_1^2$	0.1680	$\mathbf{R}_{12}$	0.1852	$\mathbf{r}_{12}$	0.2340
$\ln \bar{\mathbf{x}}_1^3$	0.1783	$\mathbf{R}_{13}$	0.2241	$\mathbf{r}_{13}$	0.2329
$\ln \bar{\mathbf{x}}_2^1$	0.1946	$\mathbf{R}_{21}$	0.2070	$\mathbf{r}_{21}$	0.5091
$\ln \bar{\mathbf{x}}_2^2$	0.1424	$\mathbf{R}_{22}$	0.1677	$\mathbf{r}_{22}$	—
$\ln \bar{\mathbf{x}}_2^3$	0.1991	$\mathbf{R}_{23}$	0.1947	$\mathbf{r}_{23}$	0.3235
$\ln \bar{\mathbf{x}}_3^1$	0.1591	$\mathbf{R}_{31}$	0.2212	$\mathbf{r}_{31}$	0.3039
$\ln \bar{\mathbf{x}}_3^2$	0.2055	$\mathbf{R}_{32}$	0.2233	$\mathbf{r}_{32}$	0.4168
$\ln \bar{\mathbf{x}}_3^3$	0.2049	$\mathbf{R}_{33}$	0.2065	$\mathbf{r}_{33}$	—

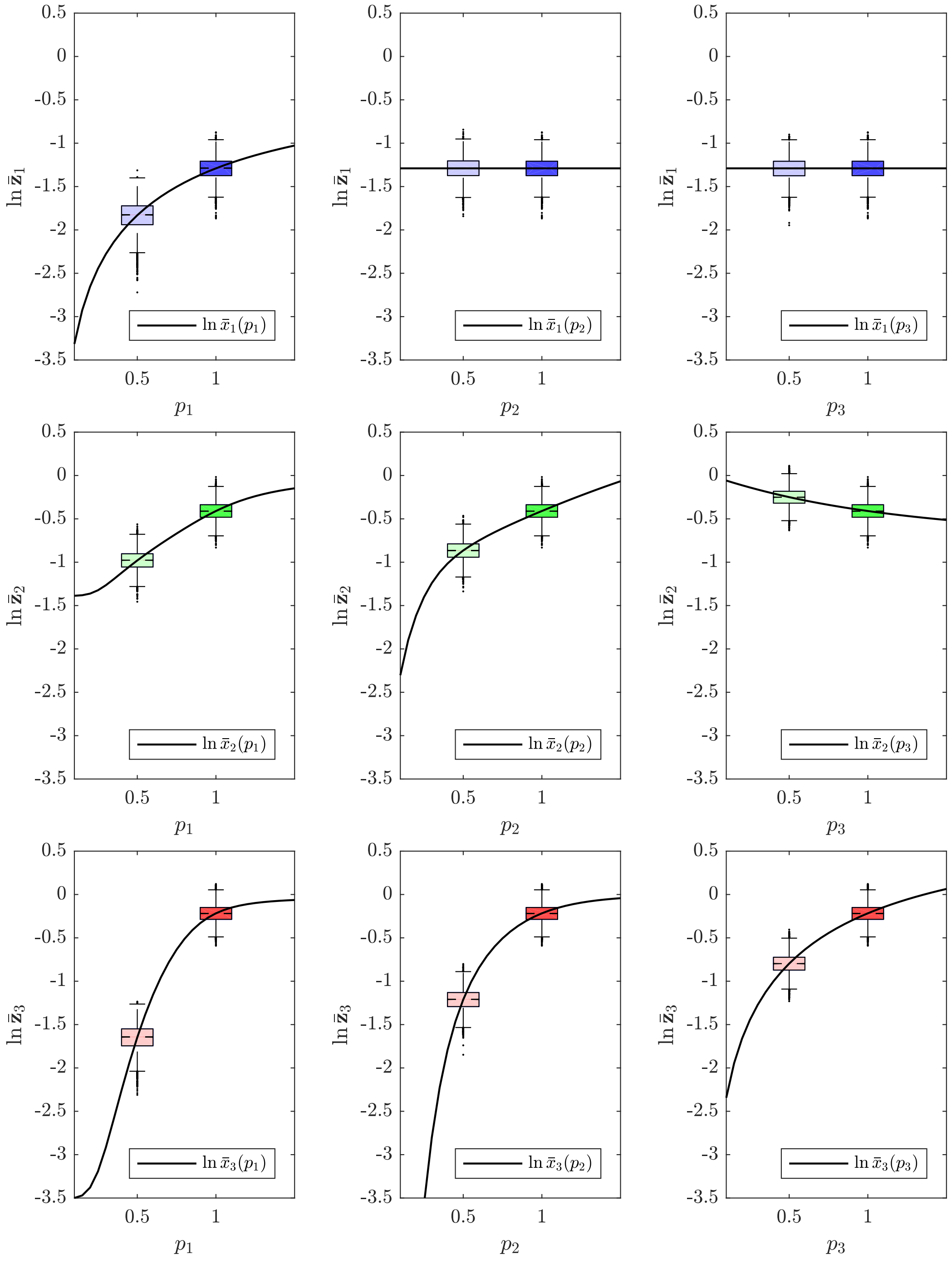

Figure S7. Nonlinear variation of the steady-state variables for changing perturbation strengths for the p53 test-bed model. The variability of the measured steady states $\bar{\mathbf{z}}_i, i = 1,2,3$ is shown in the control experiment ($p_j = 1, j = 1,2,3$) and in the 50% knockdown experiments ($p_j = 0.5, j = 1,2,3$). We generated $n = 10,000$ realizations via Monte Carlo simulations from the noise model (9) with parameters $\sigma_\eta = 0.1$ and $\sigma_\varepsilon = 0.02$. The continuous solid lines correspond to the noise free trend of the logarithm of the steady states for changing perturbation parameters ($\ln \bar{x}_i(p_j)$).

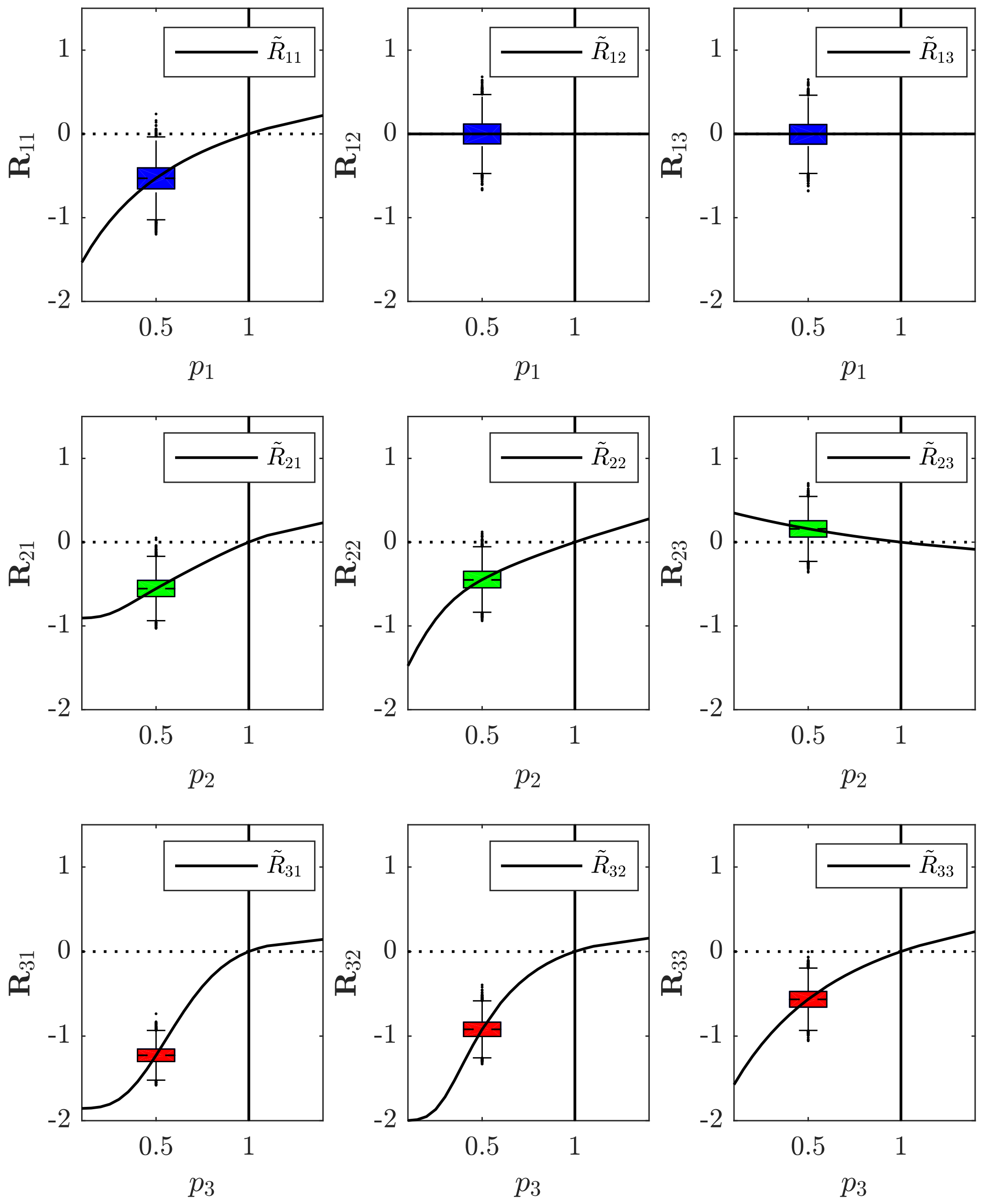

Figure S8. Nonlinear variation of the GRCs for changing perturbation strengths for the p53 test-bed model. The variability of the GRCs $\mathbf{R}_{ij}, i, j = 1,2,3$ is obtained from the sampled steady-state realizations from Fig. S7 in the case of 50% knockdown experiments ($p_j = 0.5, j = 1,2,3$). We generated $n = 10,000$ realizations via Monte Carlo simulations from the noise model (9) with parameters $\sigma_\eta = 0.1$ and $\sigma_\varepsilon = 0.02$. The continuous solid lines correspond to the noise free trend of the approximation of the GRCs for changing perturbation parameters ($\tilde{R}_{ij}(p_j)$).

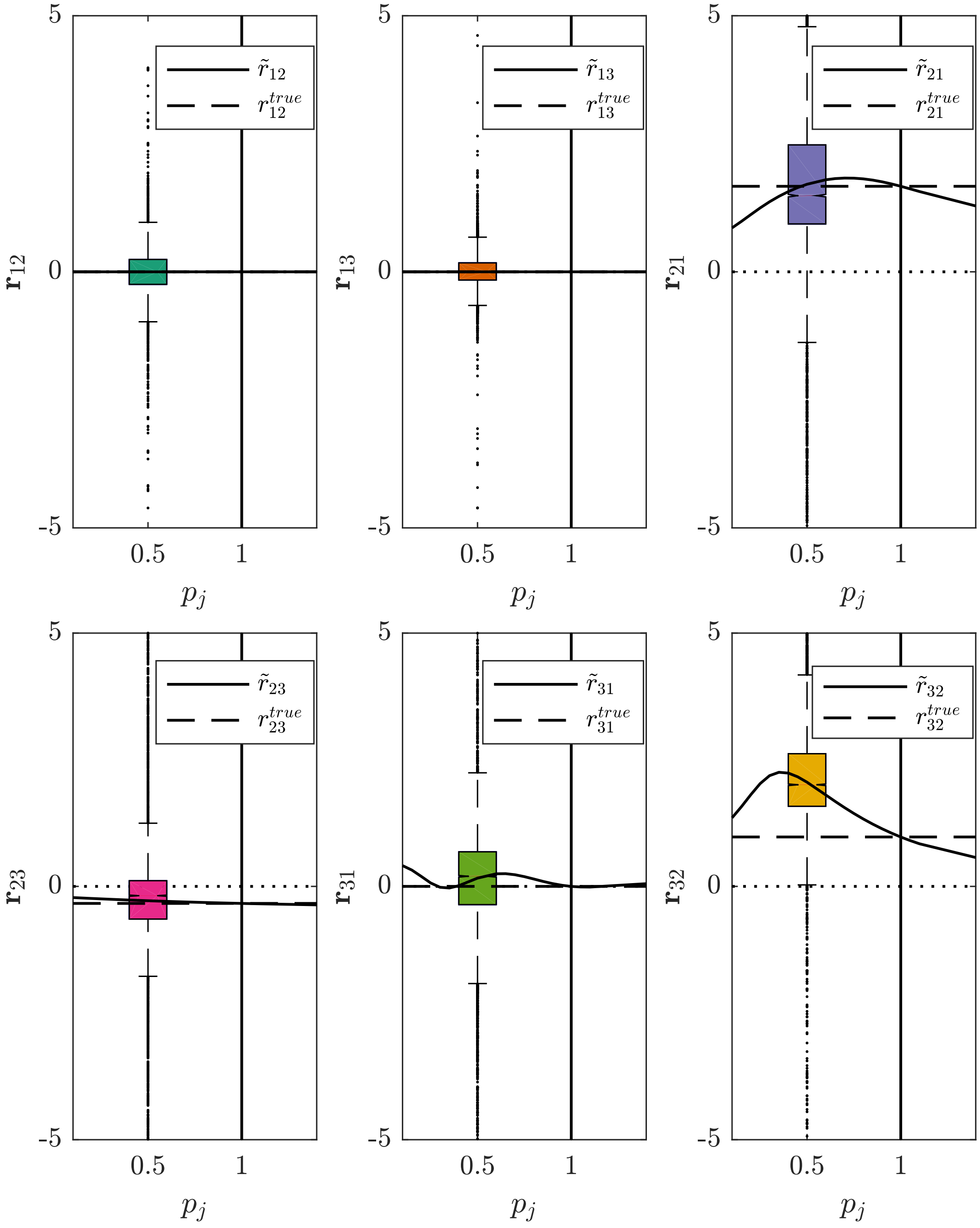

Figure S9. Nonlinear variation of the LRCs for changing perturbation strengths for the p53 test-bed model. The variability of the LRCs $\mathbf{r}_{ij}, i, j = 1,2,3$ is obtained from the sampled steady-state realizations from Fig. S7 in the case of 50% knockdown experiments ($p_j = 0.5, j = 1,2,3$). We generated $n = 10,000$ realizations via Monte Carlo simulations from the noise model (9) with parameters $\sigma_\eta = 0.1$ and $\sigma_\varepsilon = 0.02$. The continuous solid lines correspond to the noise free trend of the approximation of the LRCs for changing perturbation parameters ($\tilde{r}_{ij}(p_j)$).

S4 - Experiment design: choice of the perturbation strength

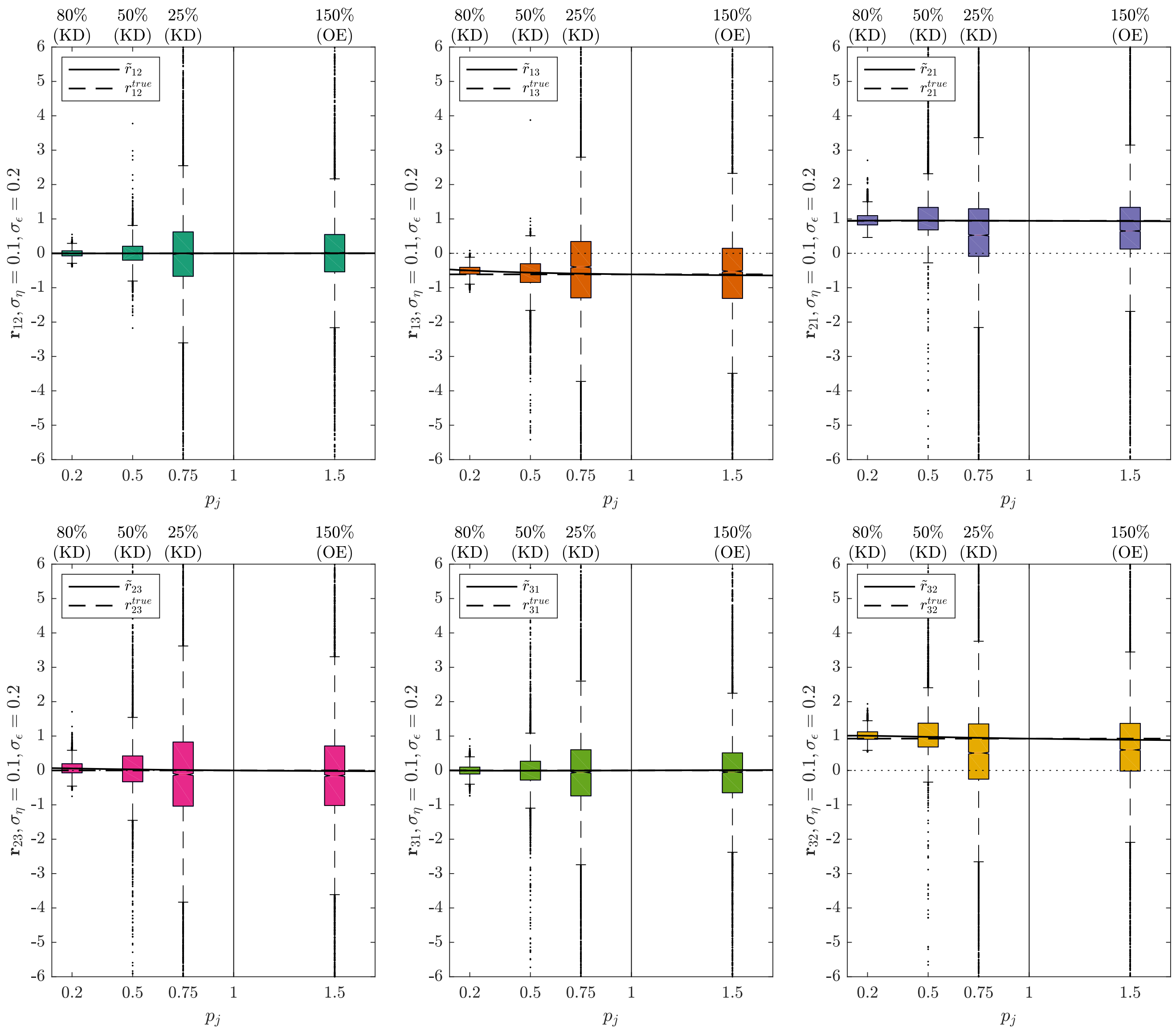

Figure S10. **Effects of different perturbation strengths on MRA based network reconstruction for the MAPK test-bed model.** Comparison of the boxplots of the estimated coefficients $\mathbf{r}_{ij}, i, j = 1, 2, 3, i \neq j$, for different perturbation strengths: 80%, 50%, 25% knockdowns (KD) and 150% overexpression (OE) of the total protein concentrations. These distributions were obtained from $n = 10,000$ realizations via Monte Carlo simulations for plausible input noise levels of both multiplicative and additive components: $\sigma_\eta = 0.1$ and $\sigma_\varepsilon = 0.2$.

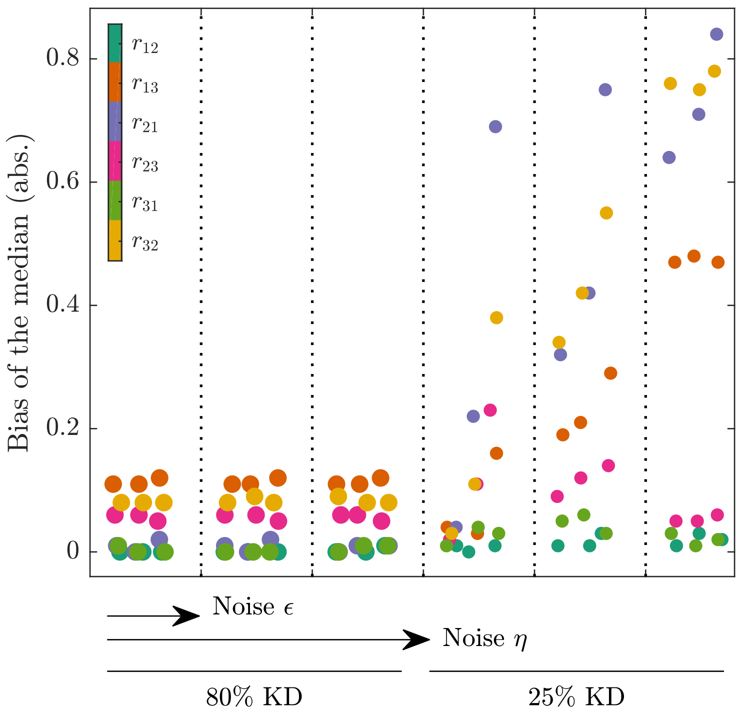

Figure S11. Effects of different perturbation strengths on MRA based network reconstruction for the MAPK test-bed model. Comparison of the behaviour of the bias of the median for large and small perturbations for increasing levels of multiplicative and additive noise: $\sigma_\eta \in \{0.05, 0.1, 0.2\}$ and $\sigma_\varepsilon \in \{0.1, 0.2, 0.5\}$.

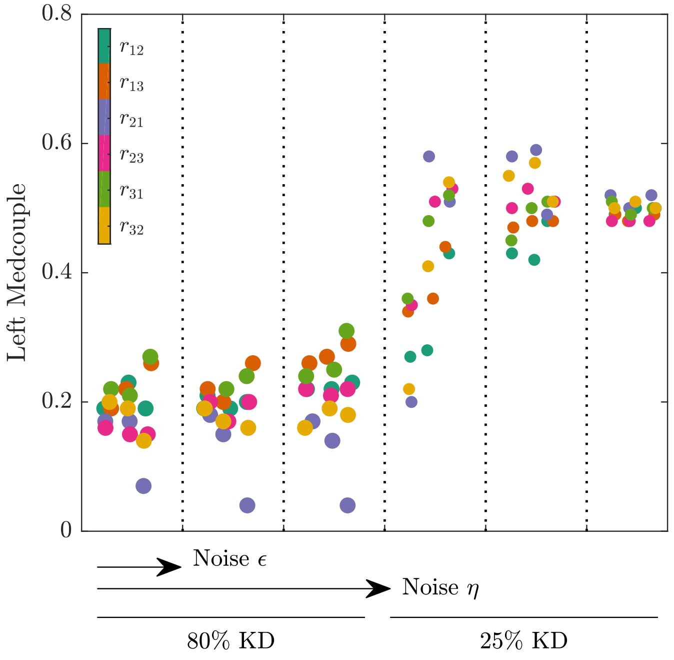

(a) Left medcouple

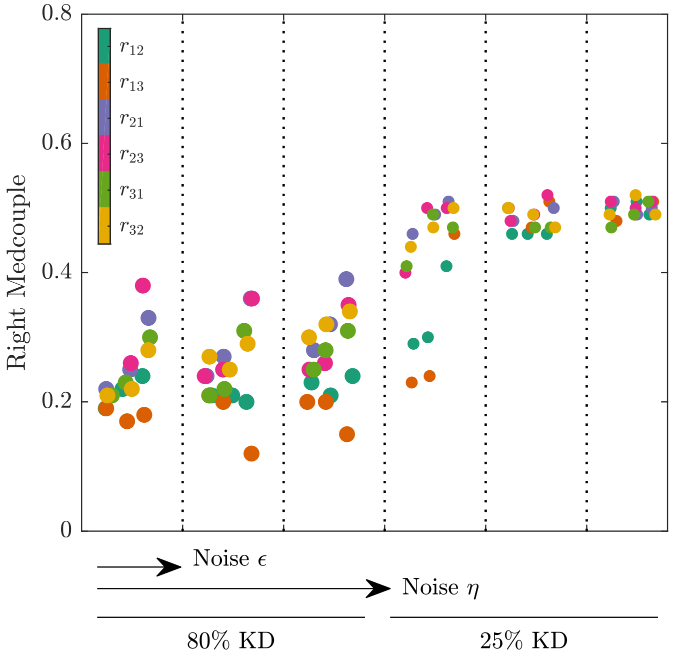

(b) Right medcouple

Figure S12. Effects of different perturbation strengths on MRA based network reconstruction for the MAPK test-bed model. Comparison of the behavior of **(a)** left and **(b)** right medcouples for large and small perturbations for increasing levels of multiplicative and additive noise: $\sigma_\eta \in \{0.05, 0.1, 0.2\}$ and $\sigma_\varepsilon \in \{0.1, 0.2, 0.5\}$.

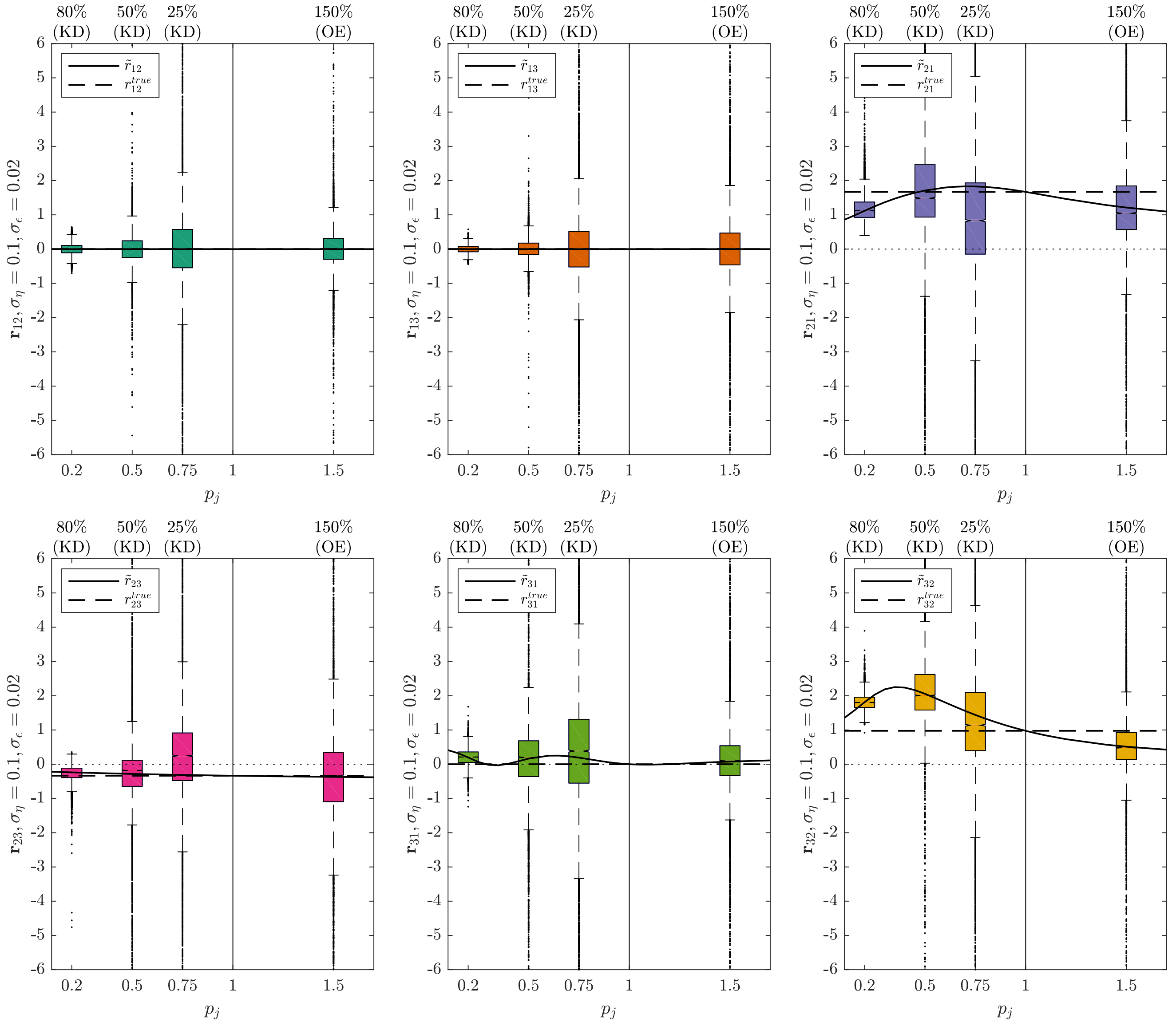

Figure S13. Effects of different perturbation strengths on MRA based network reconstruction for the p53 test-bed model. Comparison of the boxplots of the estimated coefficients $\mathbf{r}_{ij}, i, j = 1, 2, 3, i \neq j$, for different perturbation strengths: 80%, 50%, 25% knockdowns (KD) and 150% overexpression (OE) of the total protein concentrations. These distributions were obtained from $n = 10,000$ realizations via Monte Carlo simulations for plausible input noise levels of both multiplicative and additive components: $\sigma_\eta = 0.1$ and $\sigma_\varepsilon = 0.02$.

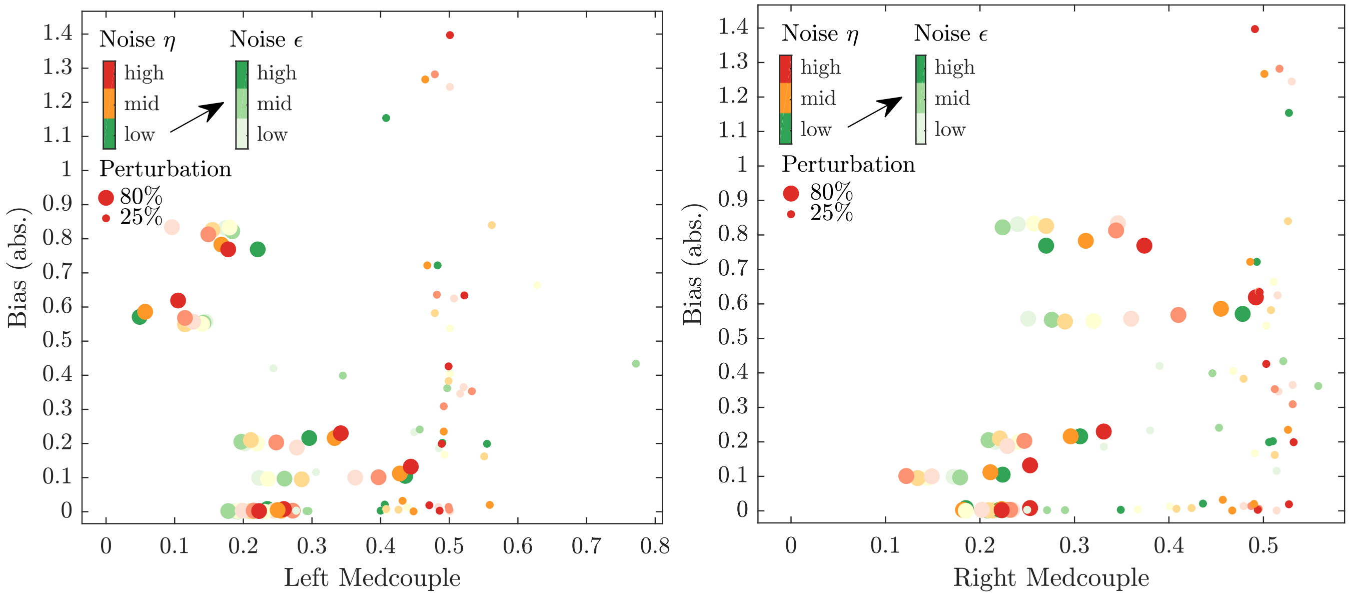

(a) Bias over Left medcouple **(b)** Bias over Right medcouple

Figure S14. Effects of different perturbation strengths on MRA based network reconstruction for the p53 test-bed model. Visualization of bias of the median over **(a)** left and **(b)** right medcouples of the distributions of all LRC obtained with large (80%) or small (25%) knockdown strength of the total protein concentrations. These statistics are given for increasing levels of multiplicative and additive noise: $\sigma_\eta \in \{0.05, 0.1, 0.2\}$ and $\sigma_\varepsilon \in \{0.01, 0.02, 0.05\}$.

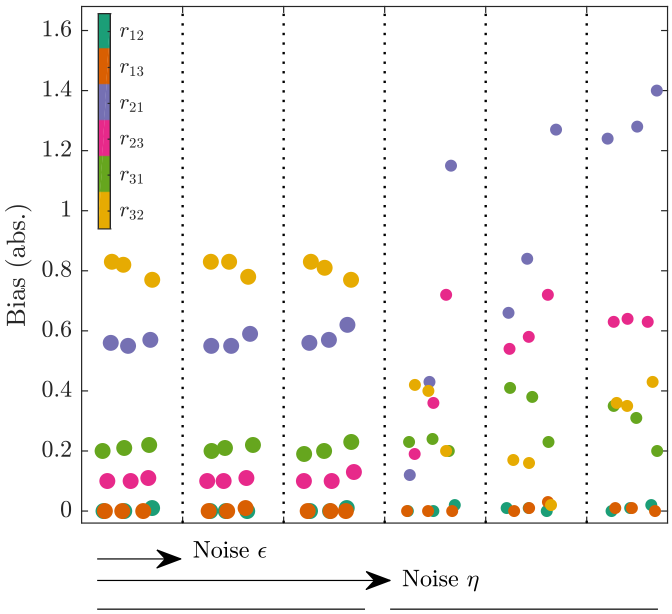

Figure S15. Effects of different perturbation strengths on MRA based network reconstruction for the p53 test-bed model. Comparison of the behaviour of the bias of the median for large and small perturbations for increasing levels of multiplicative and additive noise: $\sigma_\eta \in \{0.05, 0.1, 0.2\}$ and $\sigma_\varepsilon \in \{0.01, 0.02, 0.05\}$.

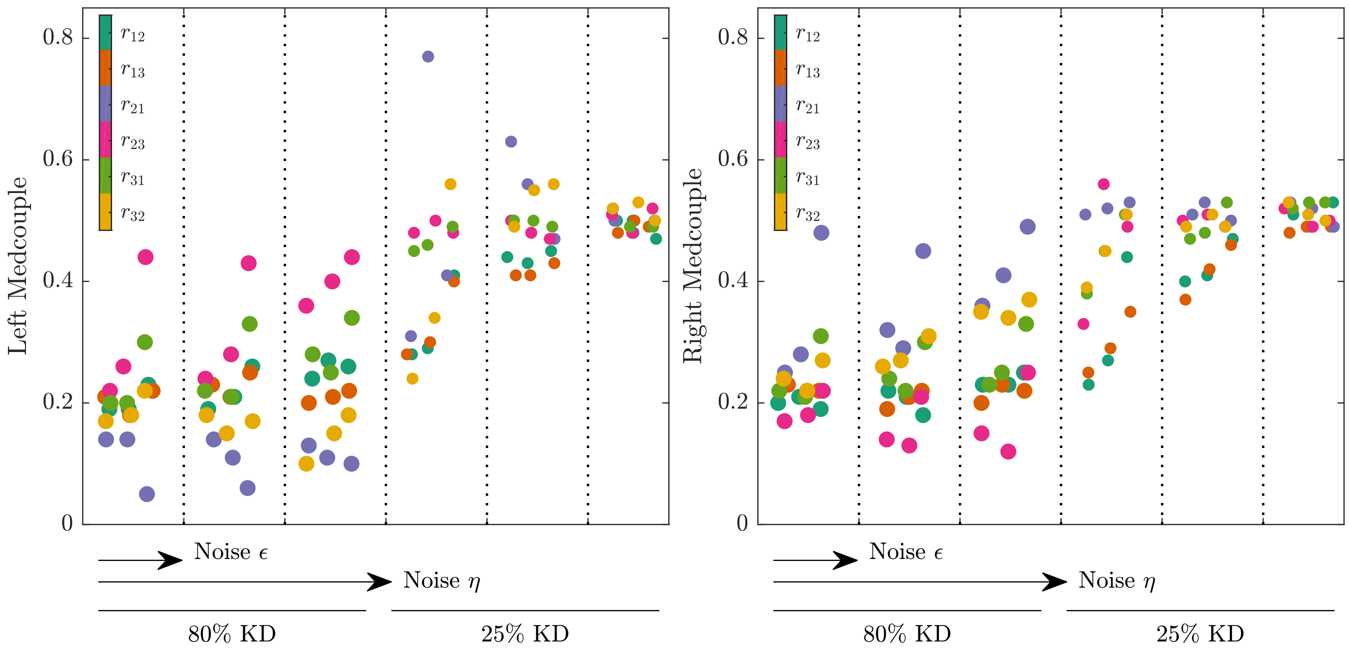

(a) Left medcouple **(b)** Right medcouple

Figure S16. Effects of different perturbation strengths on MRA based network reconstruction for the p53 test-bed model. Comparison of the behavior of **(a)** left and **(b)** right medcouples for large and small perturbations for increasing levels of multiplicative and additive noise: $\sigma_\eta \in \{0.05, 0.1, 0.2\}$ and $\sigma_\varepsilon \in \{0.01, 0.02, 0.05\}$.

S5 - Experiment design: choice of the control strategy

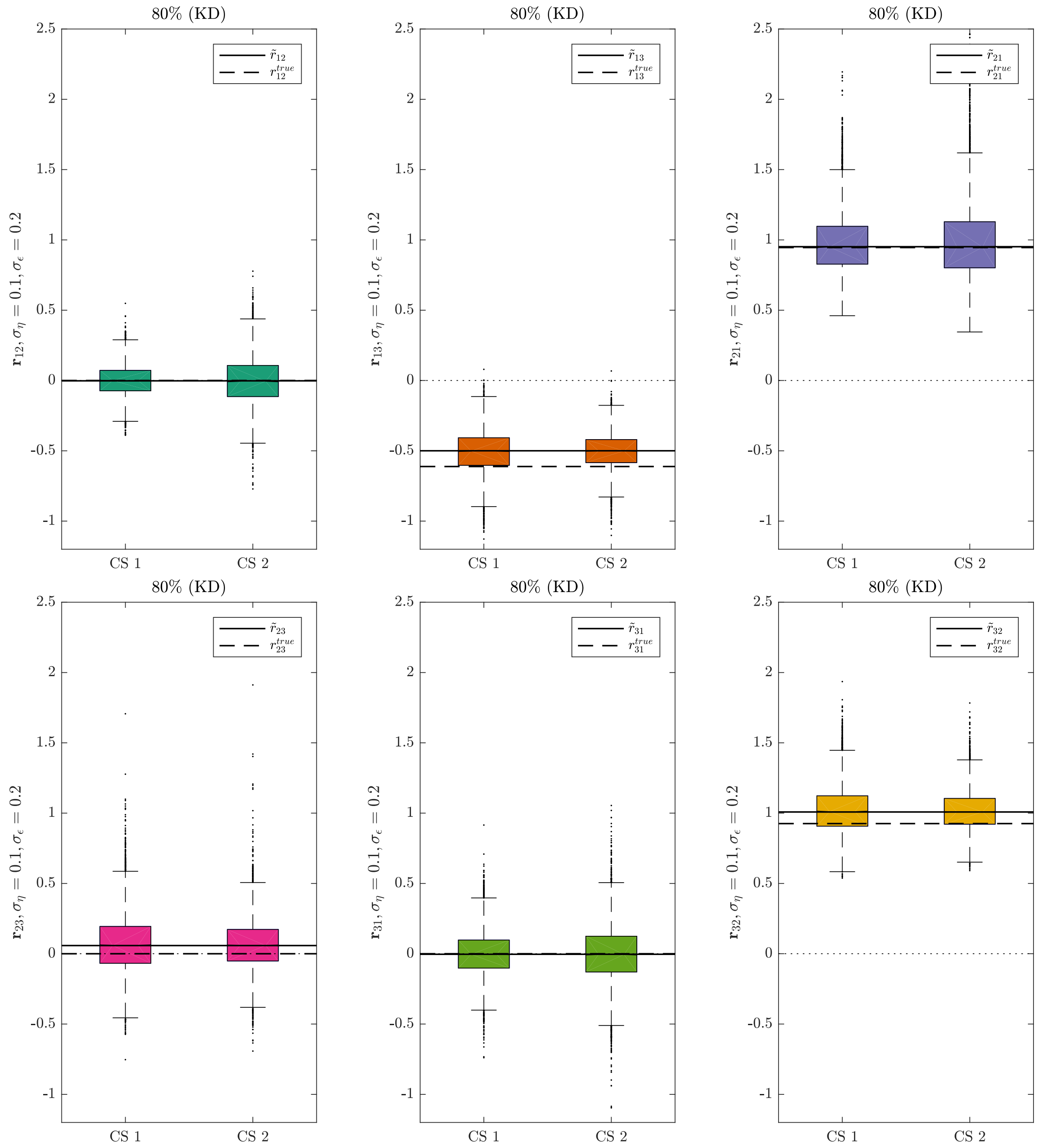

Figure S17. Effects of two alternative control strategies on MRA based network reconstruction for the MAPK test-bed model. Comparison of the boxplots of the estimated coefficients $\mathbf{r}_{ij}, i, j = 1, 2, 3, i \neq j$, for the two different control strategies CS1 and CS2. These distributions were obtained for plausible input noise levels of both multiplicative and additive components: $\sigma_\eta = 0.1$ and $\sigma_\varepsilon = 0.2$.

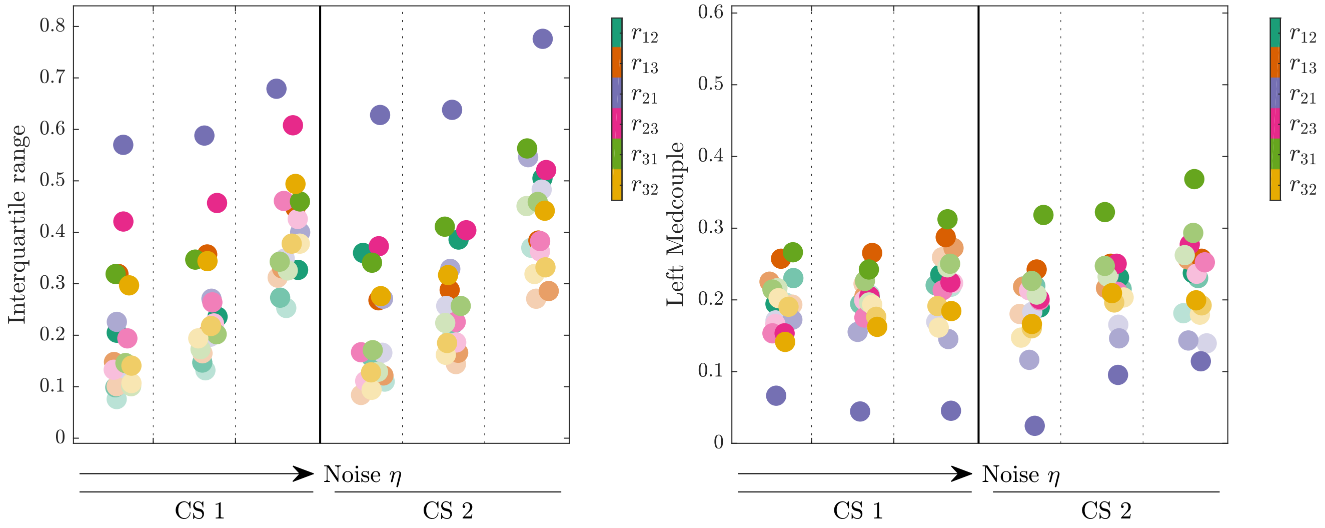

(a) Interquartile range **(b)** Left medcouple

Figure S18. Effects of two alternative control strategies on MRA based network reconstruction for the MAPK test-bed model. Comparison of the behaviour of **(a)** IQR and **(b)** left medcouples for two alternative control strategies for increasing levels of multiplicative and additive noise: $\sigma_\eta \in \{0.05, 0.1, 0.2\}$ and $\sigma_\varepsilon \in \{0.1, 0.2, 0.5\}$.

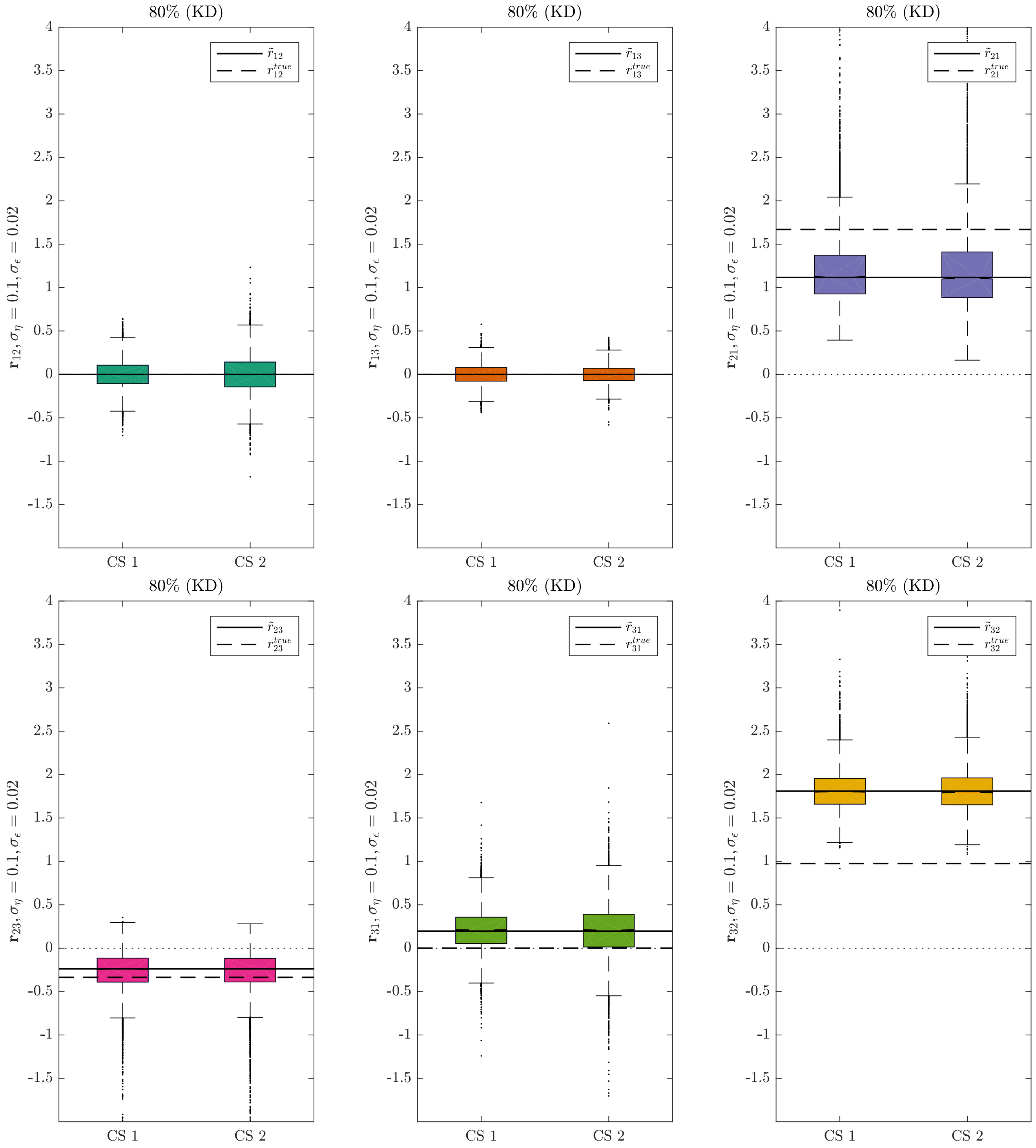

Figure S19. Effects of two alternative control strategies on MRA based network reconstruction for the p53 test-bed model. Comparison of the boxplots of the estimated coefficients $\mathbf{r}_{ij}, i, j = 1,2,3, i \neq j$, for the two different control strategies CS1 and CS2. These distributions were obtained for plausible input noise levels of both multiplicative and additive components: $\sigma_\eta = 0.1$ and $\sigma_\varepsilon = 0.02$.

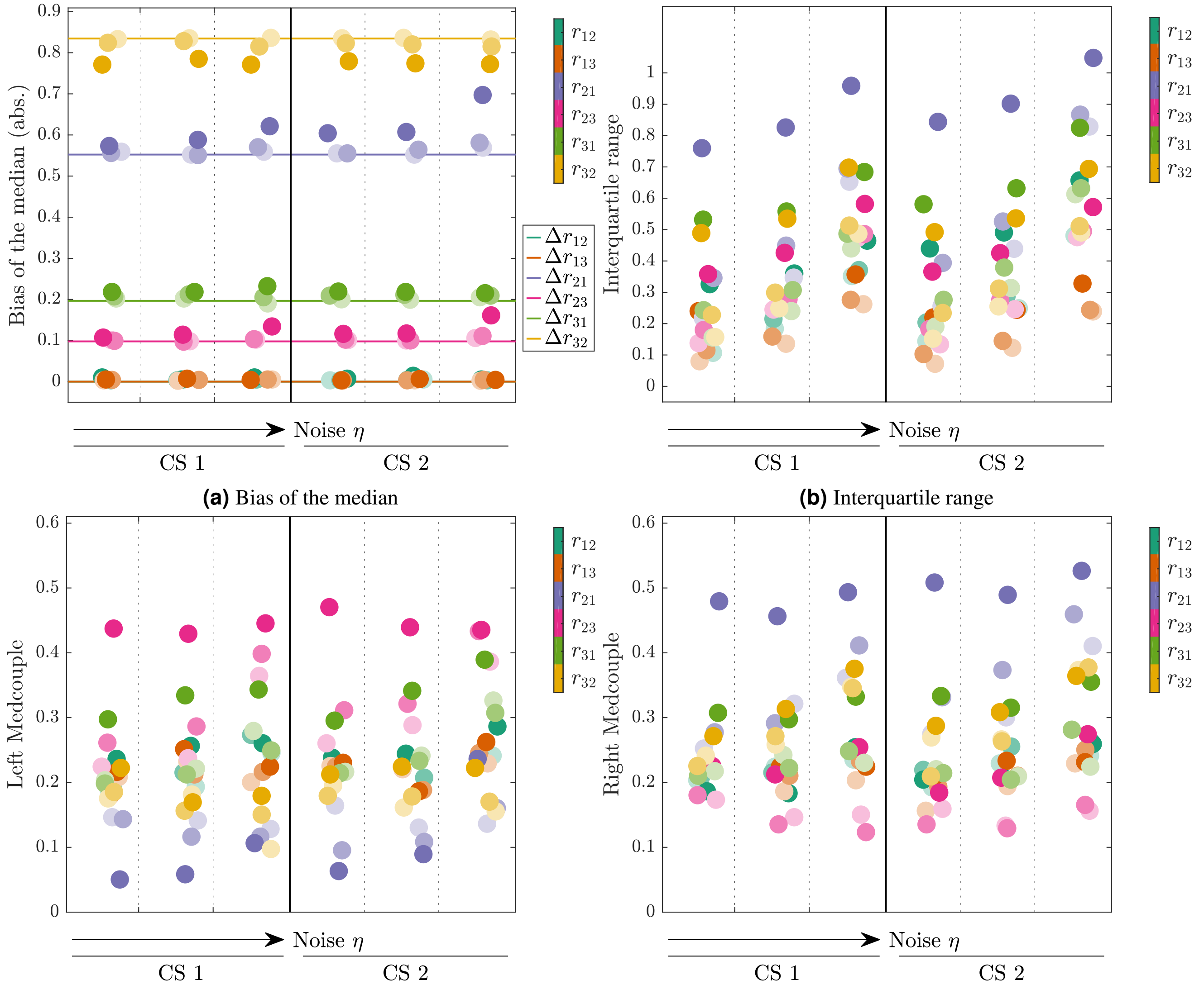

(a) Bias of the median **(b)** Interquartile range **(c)** Left medcouple **(d)** Right medcouple

Figure S20. Effects of two alternative control strategies on MRA based network reconstruction for the p53 test-bed model. Comparison of the behaviour of **(a)** the bias of the median, **(b)** IQR, **(c)** left and **(d)** right medcouples for two alternative control strategies for increasing levels of multiplicative and additive noise: $\sigma_\eta \in \{0.05, 0.1, 0.2\}$ and $\sigma_\varepsilon \in \{0.01, 0.02, 0.05\}$.

S6 - Estimation methods to handle multiple replicates and solve the linear regression problem

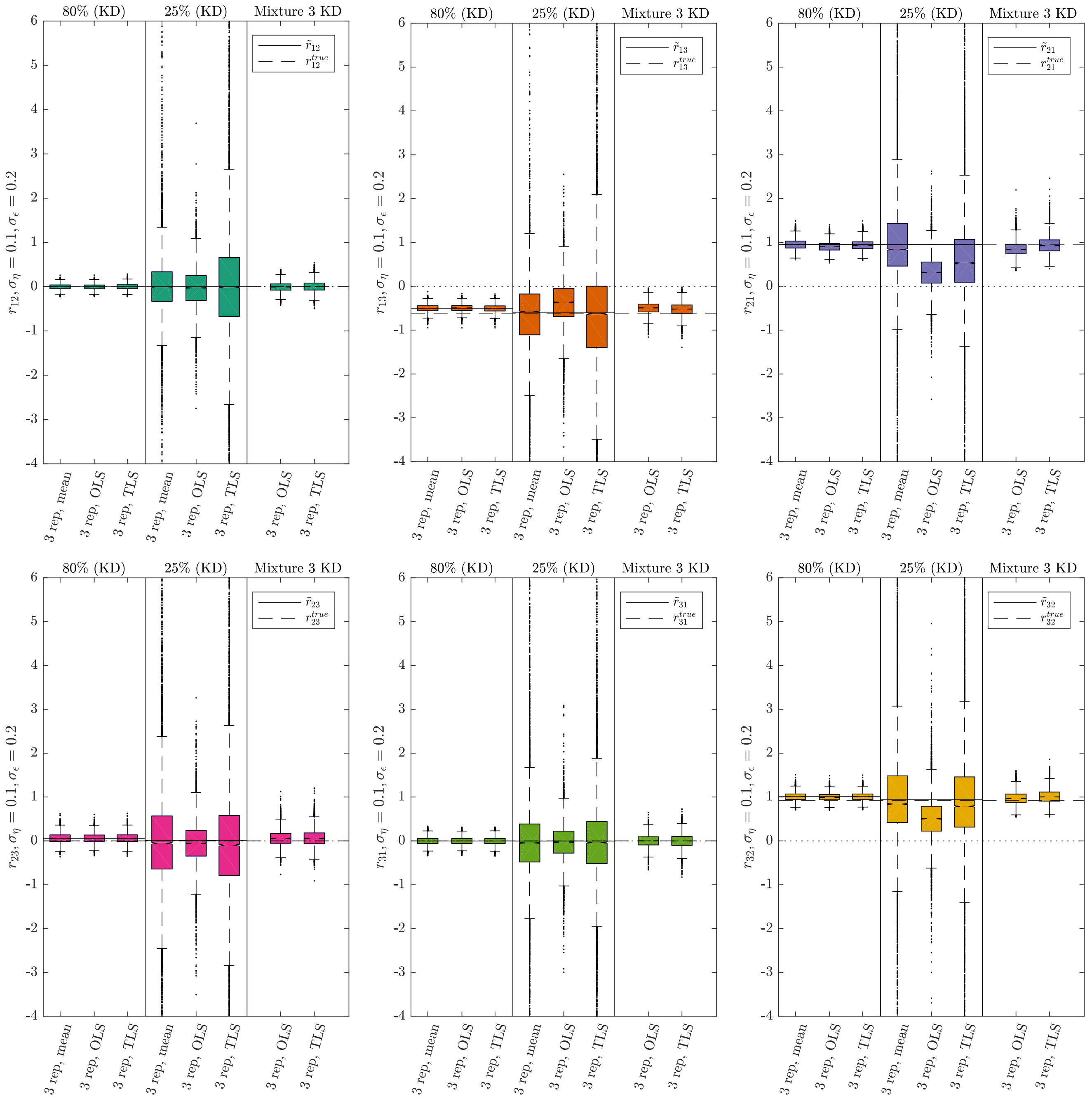

Figure S21. Effects of different estimation methods to solve the linear regression problem, in presence of more replicates data, on MRA based network reconstruction for the MAPK test-bed model. Comparison of the boxplots of the estimated coefficients $\mathbf{r}_{ij}, i, j = 1,2,3, i \neq j$, if using different estimation methods in the case of large and small perturbation experiments, with three replicates of the experimental data, as well as the mixture of three replicates from three different knockdown experiments. These distributions were obtained for plausible input noise levels of both multiplicative and additive components: $\sigma_\eta = 0.1$ and $\sigma_\varepsilon = 0.2$.

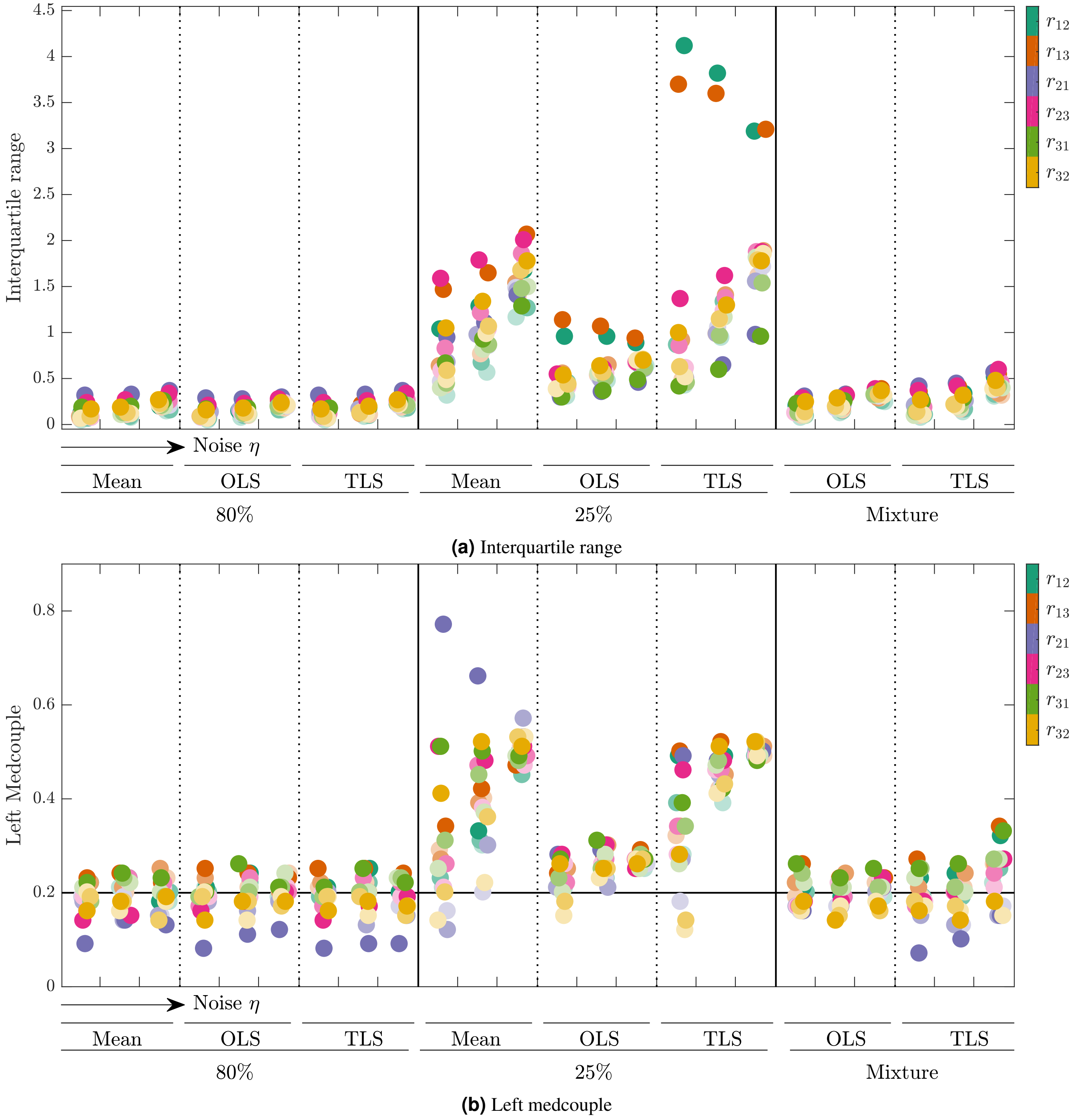

(a) Interquartile range

(b) Left medcouple

Figure S22. Effects of different estimation methods to solve the linear regression problem, in presence of more replicates data, on MRA based network reconstruction for the MAPK test-bed model. Comparison of the behavior of **(a)** IQR and **(b)** left medcouples for different numbers of replicates for increasing levels of multiplicative and additive noise: $\sigma_\eta \in \{0.05, 0.1, 0.2\}$ and $\sigma_\varepsilon \in \{0.1, 0.2, 0.5\}$.

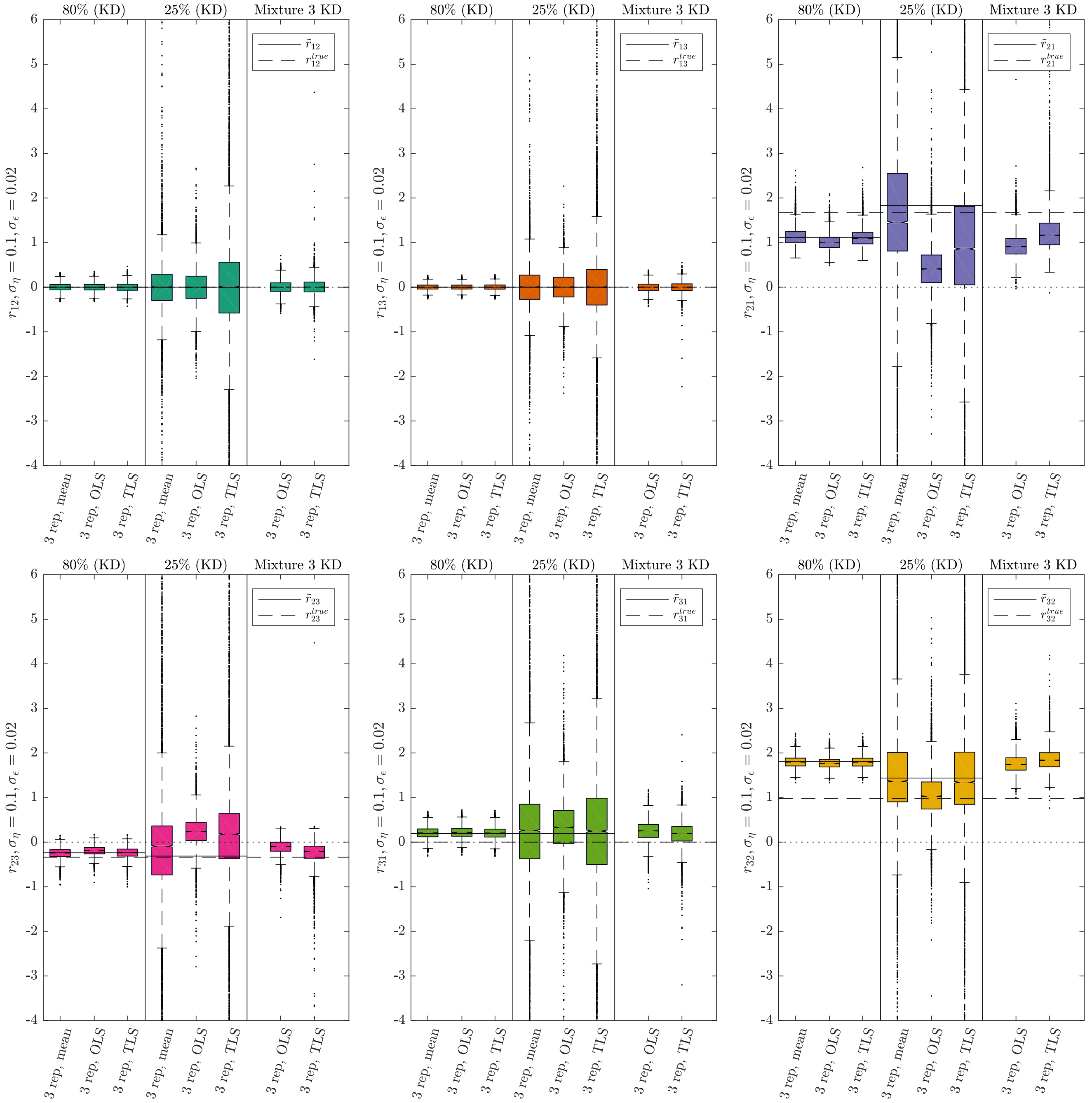

Figure S23. Effects of different estimation methods to solve the linear regression problem, in presence of more replicates data, on MRA based network reconstruction for the p53 test-bed model. Comparison of the boxplots of the estimated coefficients $\mathbf{r}_{ij}, i, j = 1,2,3, i \neq j$, if using different estimation methods in the case of large and small perturbation experiments, with three replicates of the experimental data, as well as the mixture of three replicates from three different knockdown experiments. These distributions were obtained for plausible input noise levels of both multiplicative and additive components: $\sigma_\eta = 0.1$ and $\sigma_\varepsilon = 0.02$.

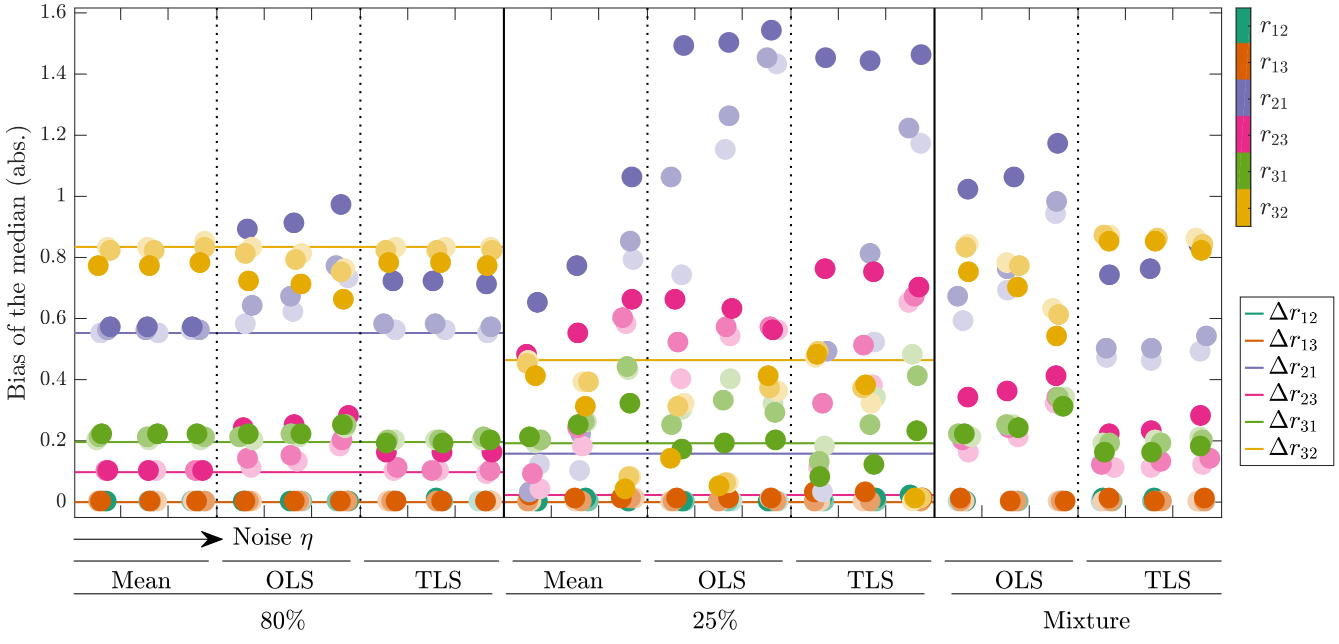

(a) Bias of the median

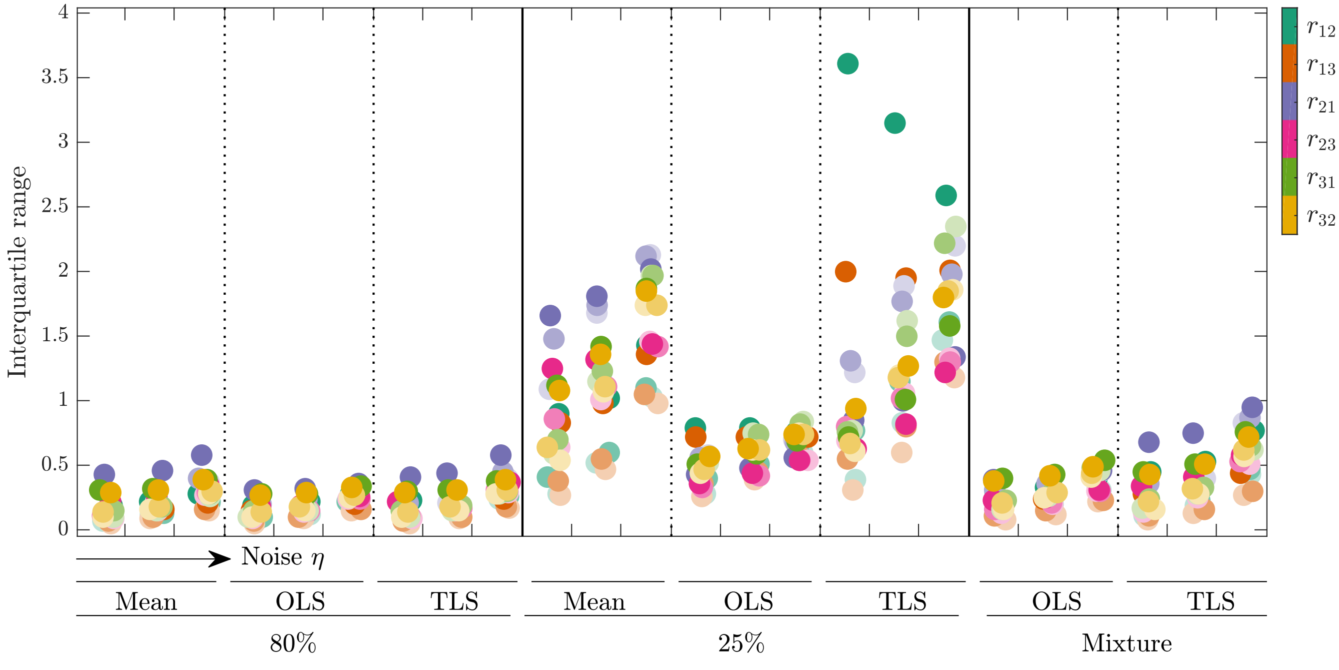

(b) Interquartile range

Figure S24. Effects of different estimation methods to solve the linear regression problem, in presence of more replicates data, on MRA based network reconstruction for the p53 test-bed model. Comparison of the behavior of **(a)** the bias of the median, **(b)** IQR, **(c)** left and **(d)** right medcouples for different numbers of technical replicates for increasing levels of multiplicative and additive noise: $\sigma_\eta \in \{0.05, 0.1, 0.2\}$ and $\sigma_\varepsilon \in \{0.01, 0.02, 0.05\}$.

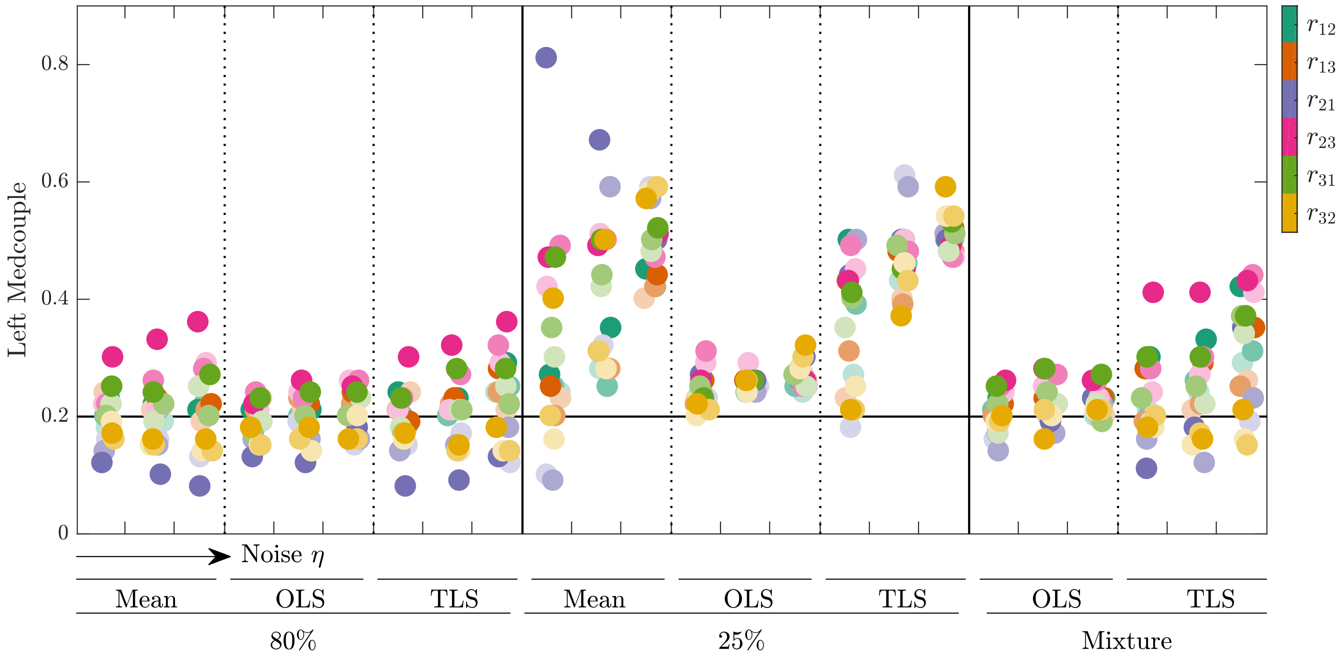

(c) Left medcouple

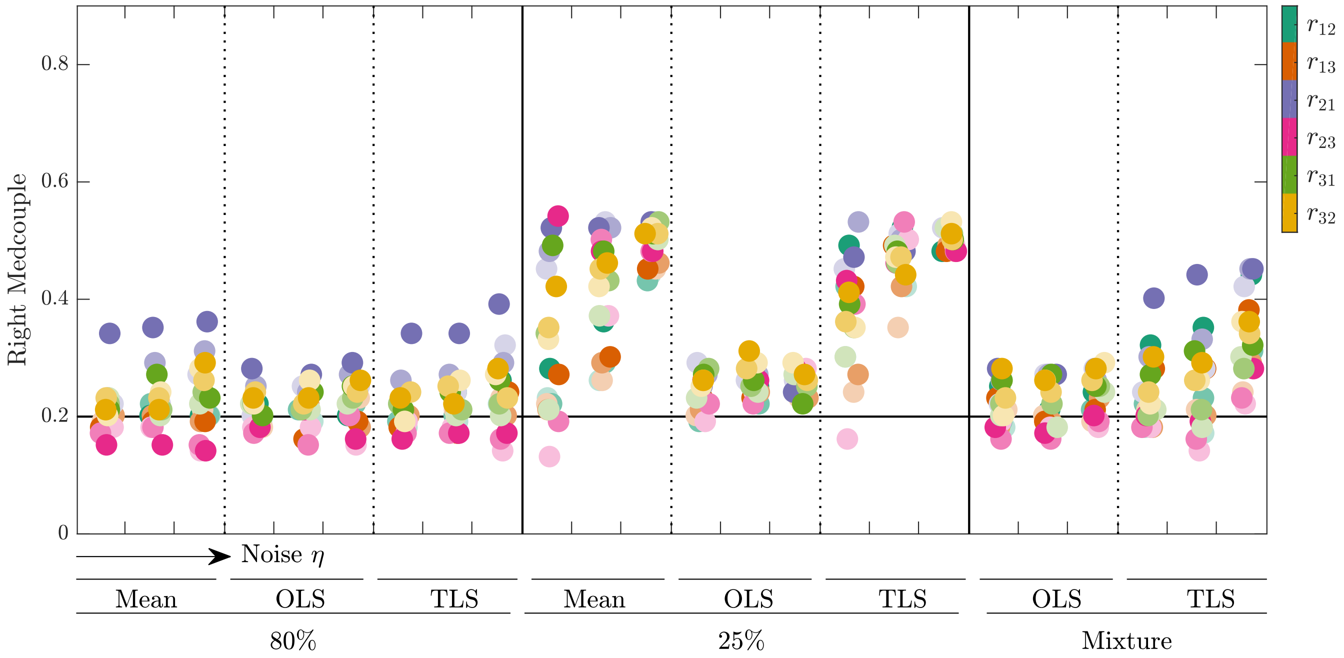

(d) Right medcouple

Figure S24. (Cont'd) Effects of different numerical solvers for the linear regression problem, in presence of more replicates data, on MRA based network reconstruction for the p53 test-bed model. Comparison of the behavior of **(a)** the bias of the median, **(b)** IQR, **(c)** left and **(d)** right medcouples for different numbers of technical replicates for increasing levels of multiplicative and additive noise: $\sigma_\eta \in \{0.05, 0.1, 0.2\}$ and $\sigma_\varepsilon \in \{0.01, 0.02, 0.05\}$.

S7 - Experiment design: number of replicates

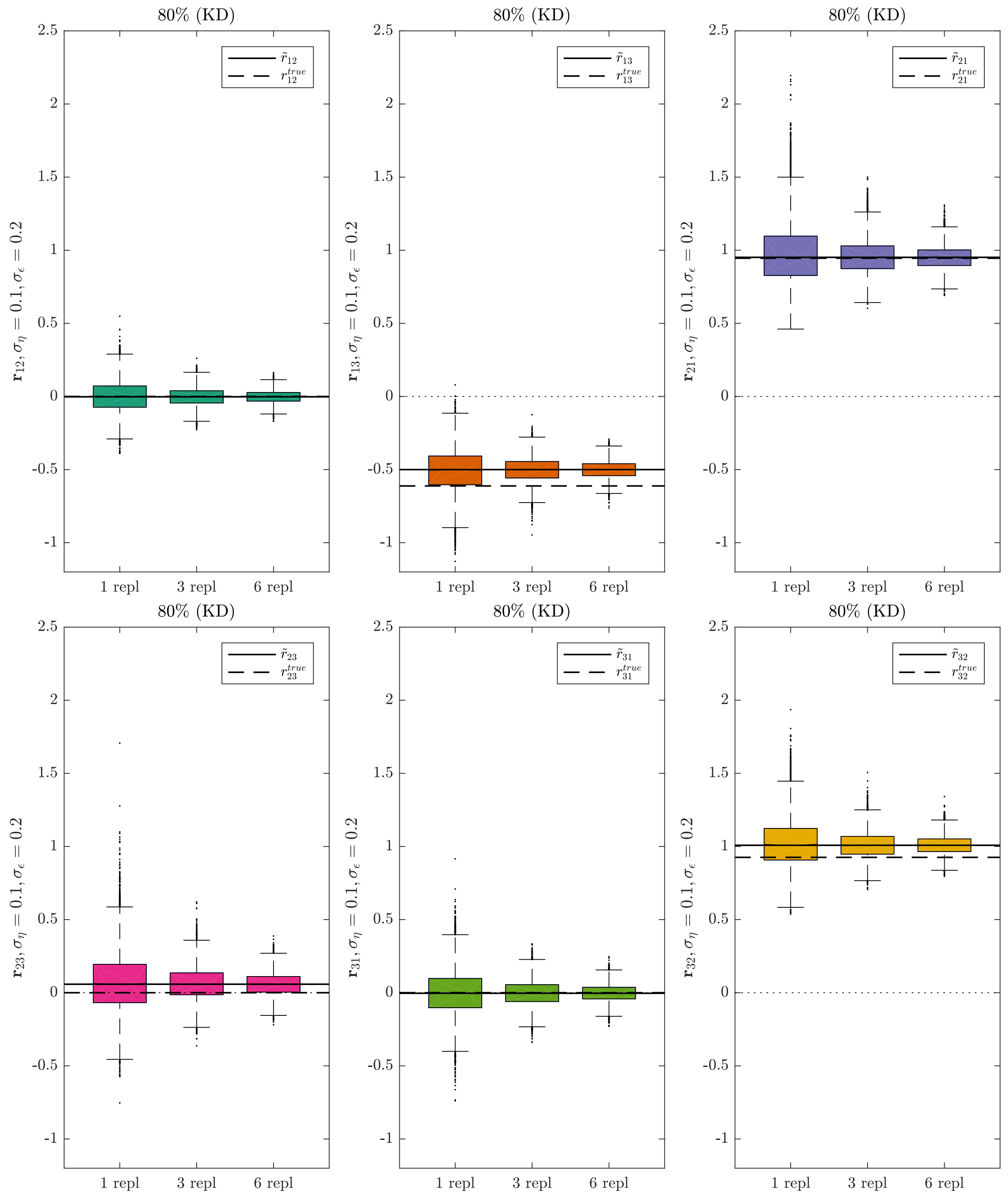

Figure S25. Effects of different numbers of replicates on MRA based network reconstruction for the MAPK test-bed model. Comparison of the boxplots of the estimated coefficients $\mathbf{r}_{ij}, i, j = 1,2,3, i \neq j$, if using different numbers of replicates of the experimental data (1, 3 and 6), and the 80% KD of total protein concentrations as perturbation experiments. These distributions were obtained for plausible input noise levels of both multiplicative and additive components: $\sigma_\eta = 0.1$ and $\sigma_\varepsilon = 0.2$.

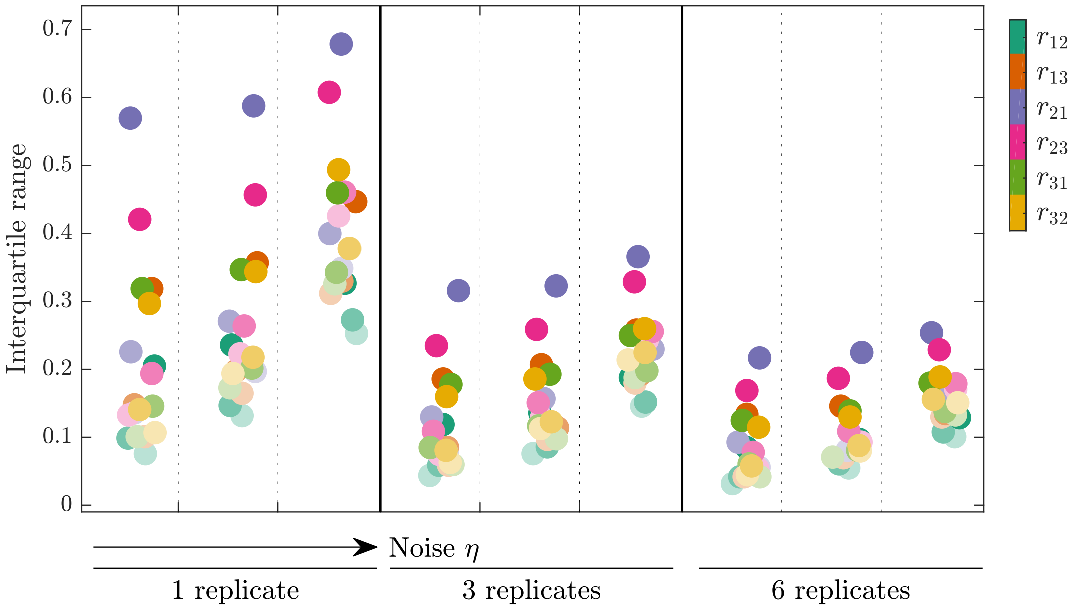

(a) Interquartile range

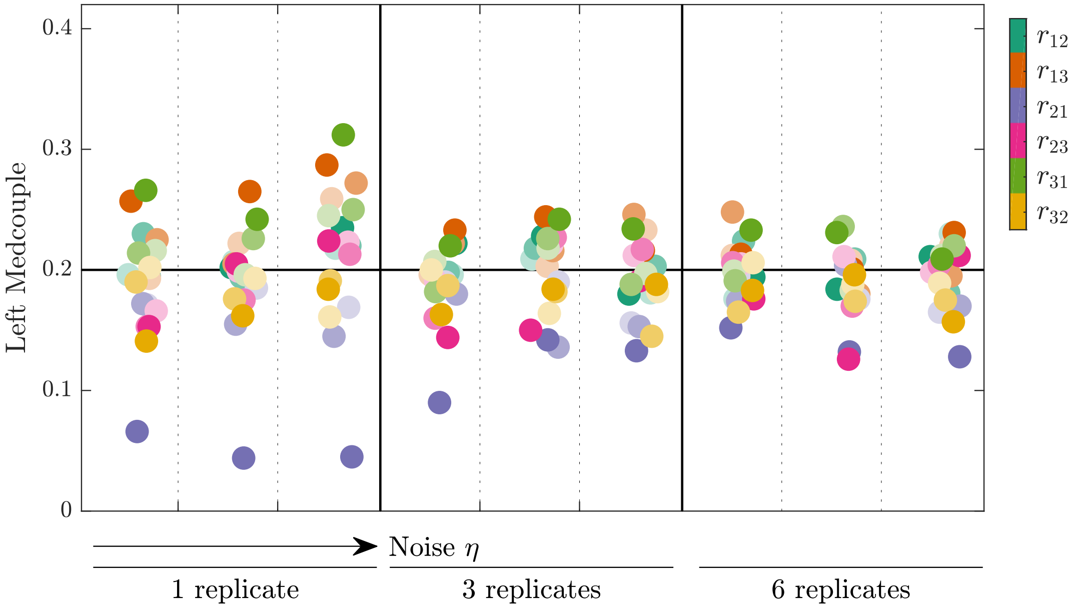

(b) Left medcouple

Figure S26. Effects of different numbers of replicates on MRA based network reconstruction for the MAPK test-bed model. Comparison of the behavior of **(a)** IQR and **(b)** left medcouples for different numbers of replicates for increasing levels of multiplicative and additive noise: $\sigma_\eta \in \{0.05, 0.1, 0.2\}$ and $\sigma_\varepsilon \in \{0.1, 0.2, 0.5\}$.

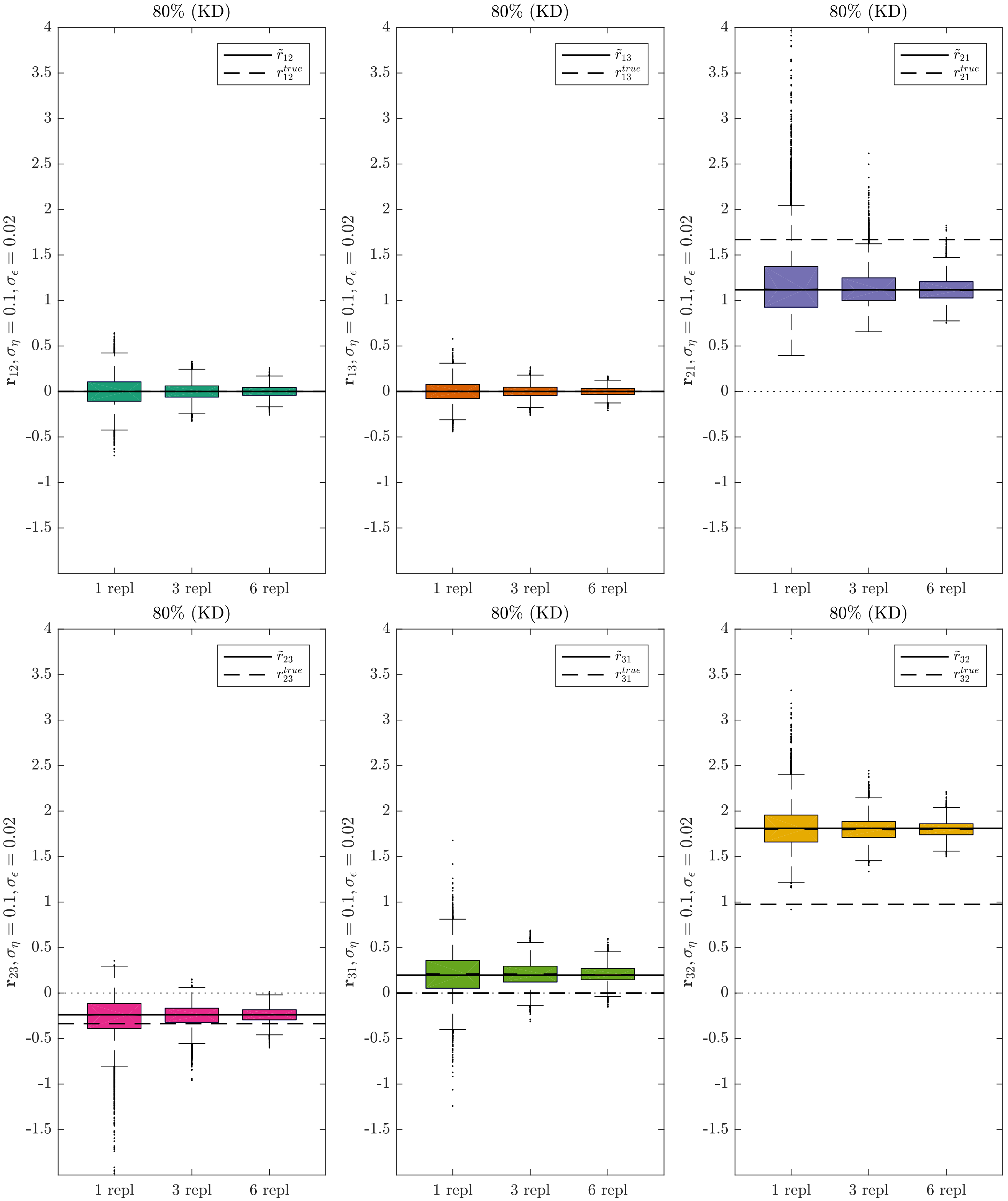

Figure S27. Effects of different numbers of replicates on MRA based network reconstruction for the p53 test-bed model. Comparison of the boxplots of the estimated coefficients $\mathbf{r}_{ij}, i, j = 1,2,3, i \neq j$, if using different numbers of replicates of the experimental data (1, 3 and 6), and the 80% KD of total protein concentrations as perturbation experiments. These distributions were obtained for plausible input noise levels of both multiplicative and additive components: $\sigma_\eta = 0.1$ and $\sigma_\varepsilon = 0.02$.

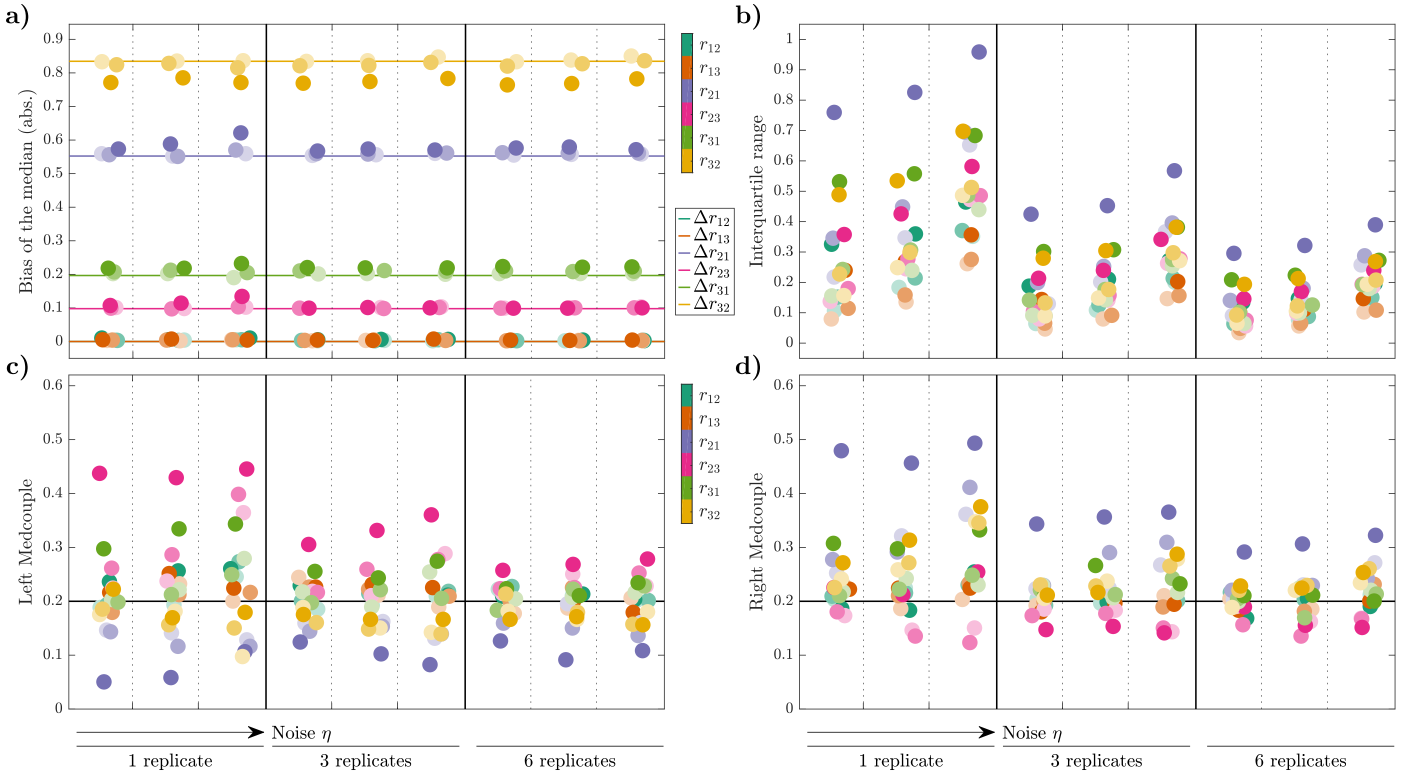

Figure S28. **Effects of different numbers of replicates on MRA based network reconstruction for the p53 test-bed model.** Comparison of the behavior of **(a)** the bias of the median, **(b)** IQR, **(c)** left and **(d)** right medcouples for different numbers of replicates for increasing levels of multiplicative and additive noise: $\sigma_\eta \in \{0.05, 0.1, 0.2\}$ and $\sigma_\varepsilon \in \{0.01, 0.02, 0.05\}$.

S8 - Performance evaluation

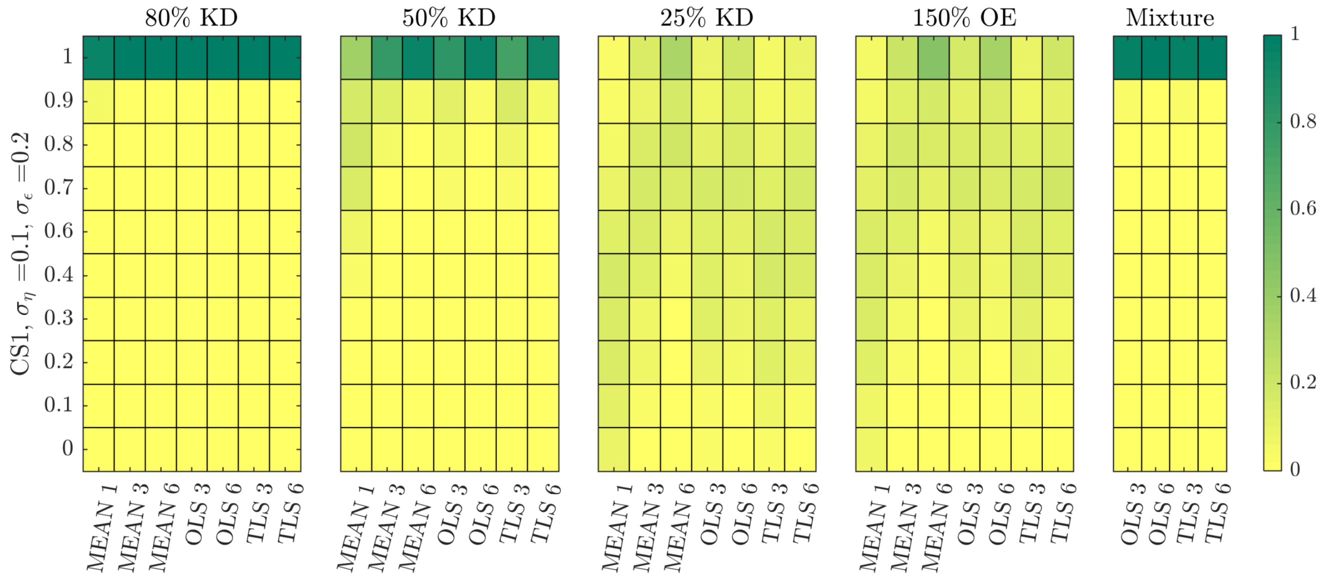

(a) Control strategy 1 and intermediate noise level: $\sigma_\eta = 0.1, \sigma_\varepsilon = 0.2$

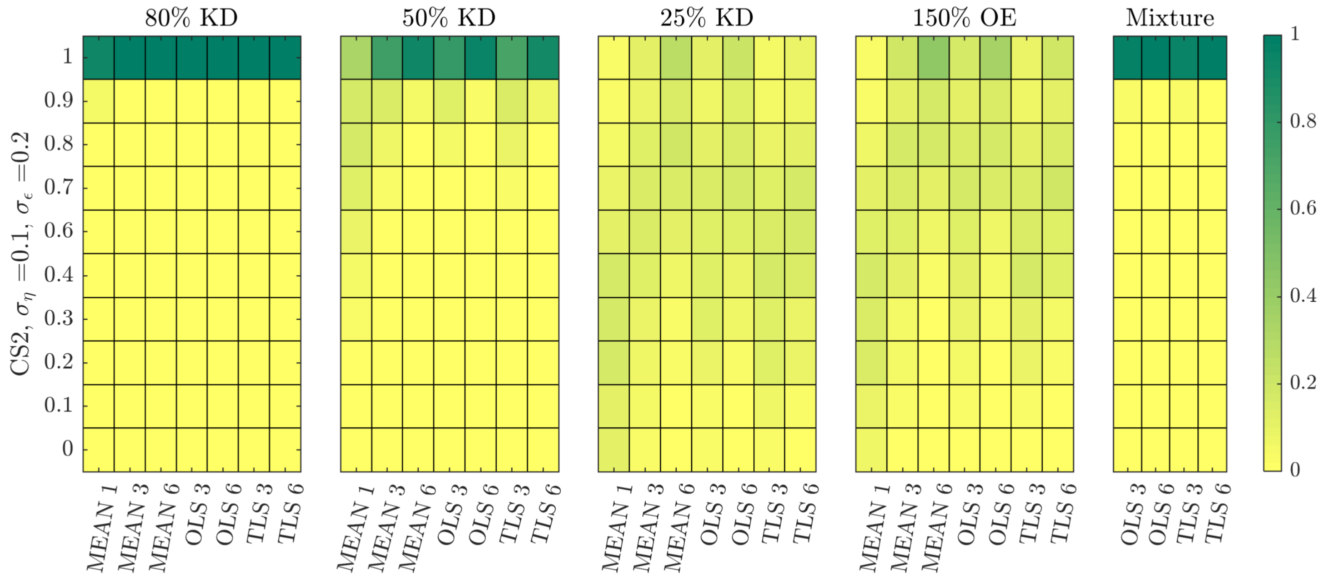

(b) Control strategy 2 and intermediate noise level: $\sigma_\eta = 0.1, \sigma_\varepsilon = 0.2$

Figure S29. Performance evaluation of all MRA formulations for network reconstruction obtained with the MAPK test-bed model. Results obtained with: **(a-b)** intermediate noise level ($\sigma_\eta = 0.1, \sigma_\varepsilon = 0.2$) and **(c-d)** high noise level ($\sigma_\eta = 0.2, \sigma_\varepsilon = 0.5$) for the two different control strategies CS1 and CS2.

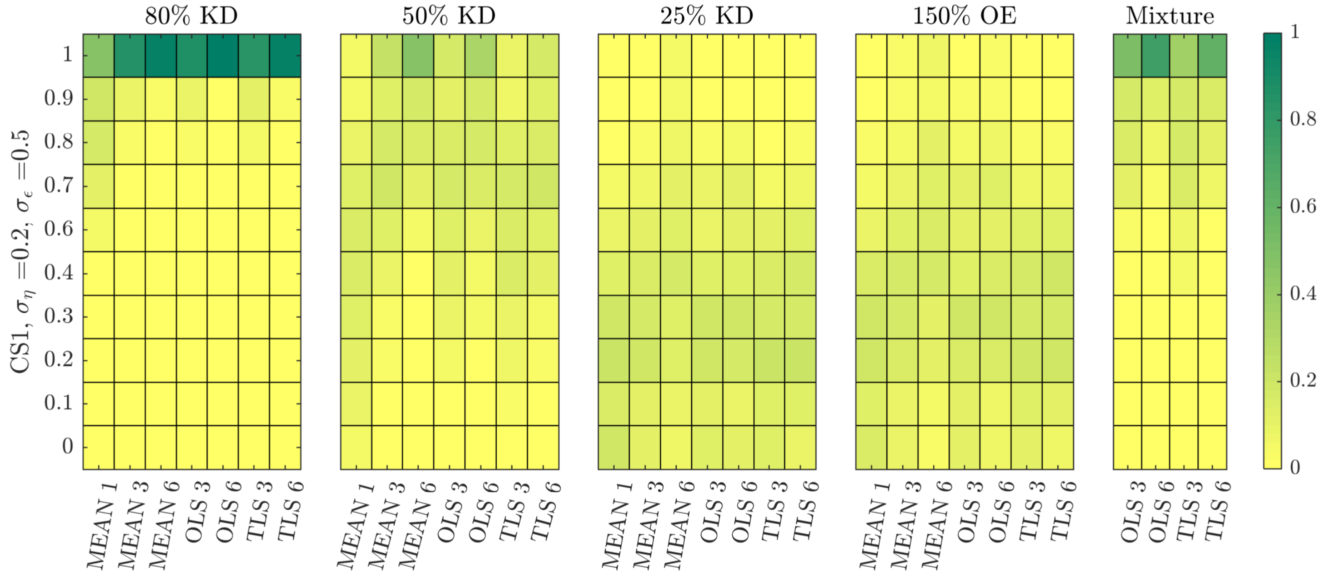

(c) Control strategy 1 and high noise level: $\sigma_\eta = 0.2, \sigma_\varepsilon = 0.5$

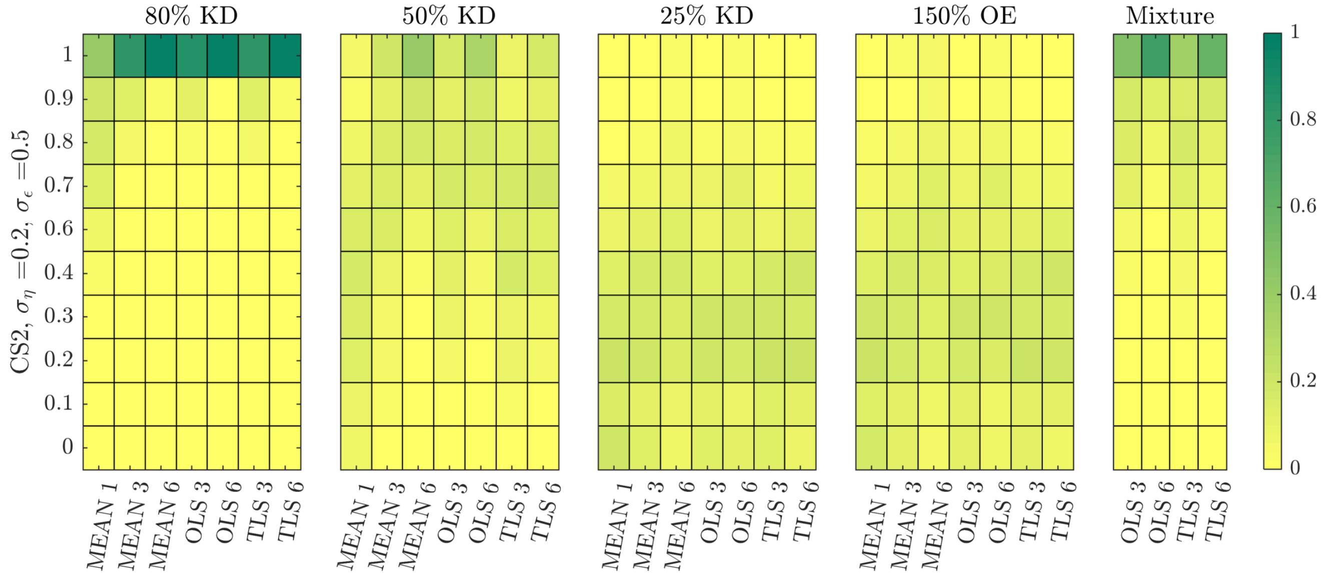

(d) Control strategy 2 and high noise level: $\sigma_\eta = 0.2, \sigma_\varepsilon = 0.5$

Figure S29. (Cont'd) Performance evaluation of all MRA formulations for network reconstruction obtained with the MAPK test-bed model. Results obtained with: **(a-b)** intermediate noise level ($\sigma_\eta = 0.1, \sigma_\varepsilon = 0.2$) and **(c-d)** high noise level ($\sigma_\eta = 0.2, \sigma_\varepsilon = 0.5$) for the two different control strategies CS1 and CS2.

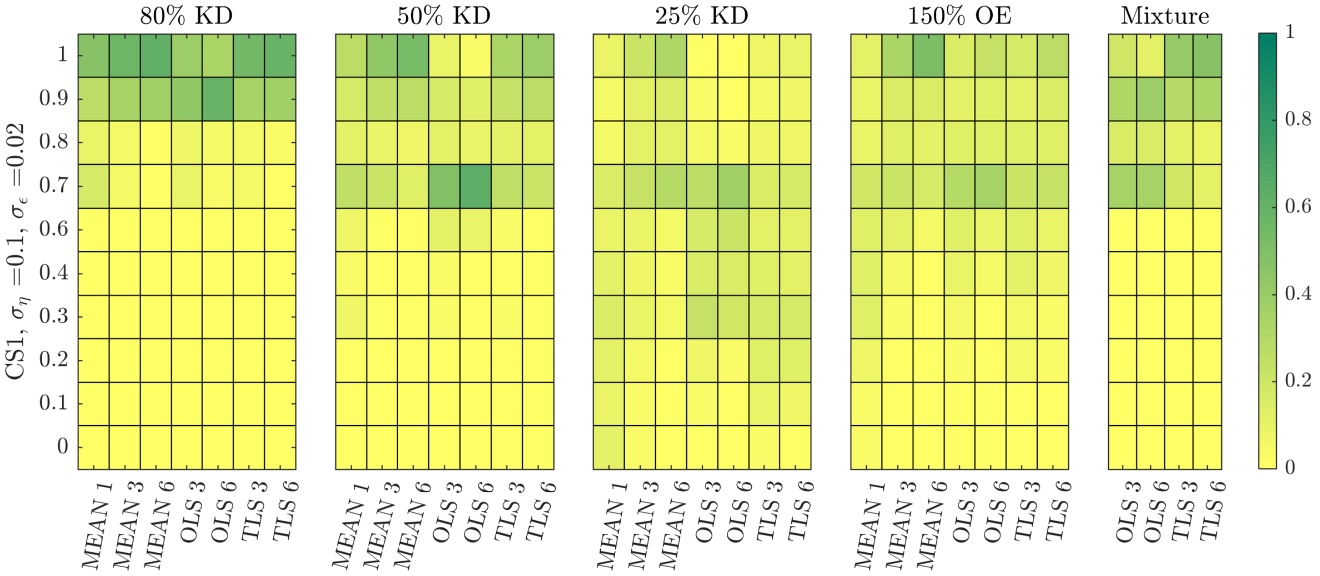

(a) Control strategy 1 and intermediate noise level: $\sigma_\eta = 0.1, \sigma_\varepsilon = 0.02$

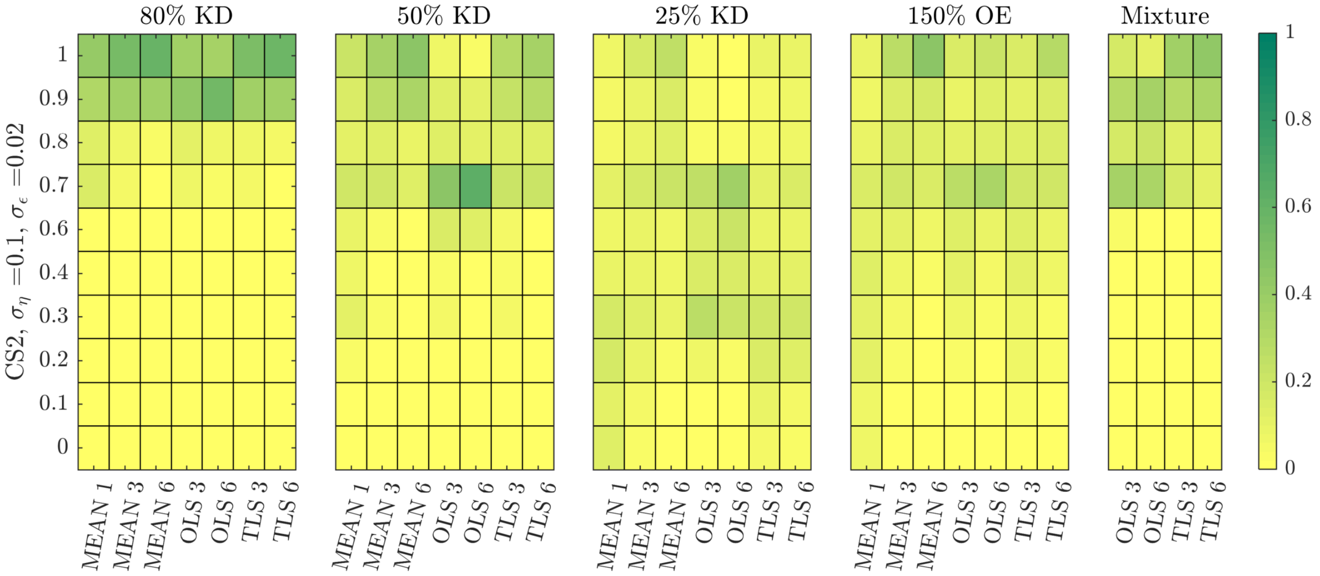

(b) Control strategy 2 and intermediate noise level: $\sigma_\eta = 0.1, \sigma_\varepsilon = 0.02$

Figure S30. Performance evaluation of all MRA formulations for network reconstruction obtained with the p53 test-bed model. Results obtained with: **(a-b)** intermediate noise level ($\sigma_\eta = 0.1, \sigma_\varepsilon = 0.02$) and **(c-d)** high noise level ($\sigma_\eta = 0.2, \sigma_\varepsilon = 0.05$) for the two different control strategies CS1 and CS2.

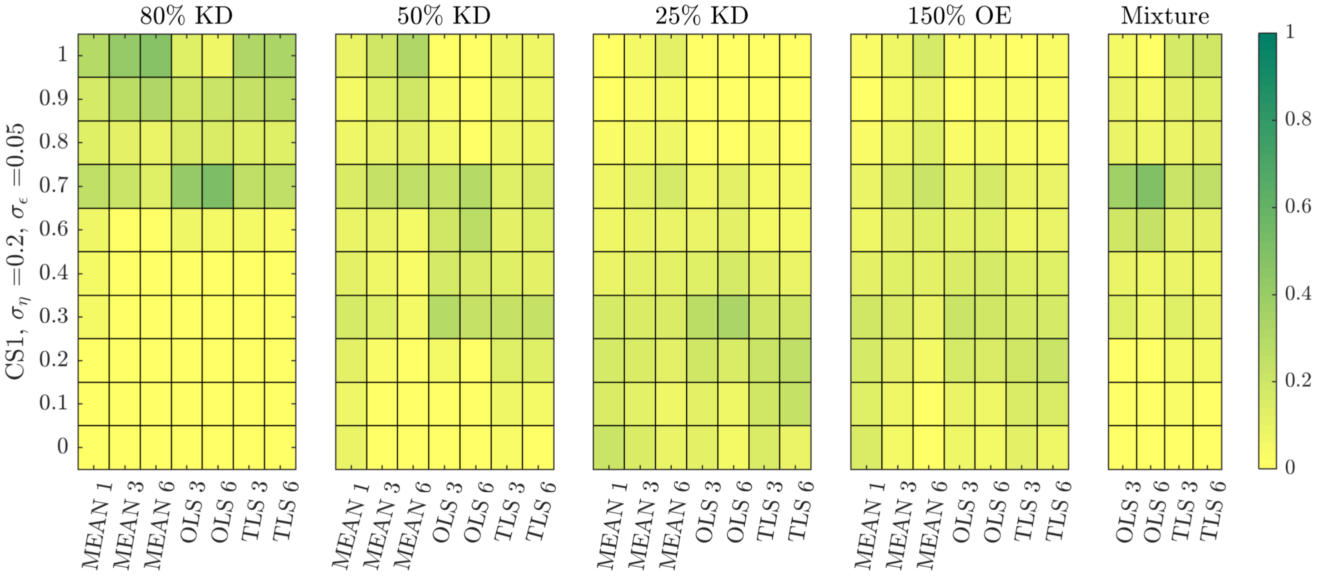

(c) Control strategy 1 and high noise level: $\sigma_\eta = 0.2, \sigma_\varepsilon = 0.05$

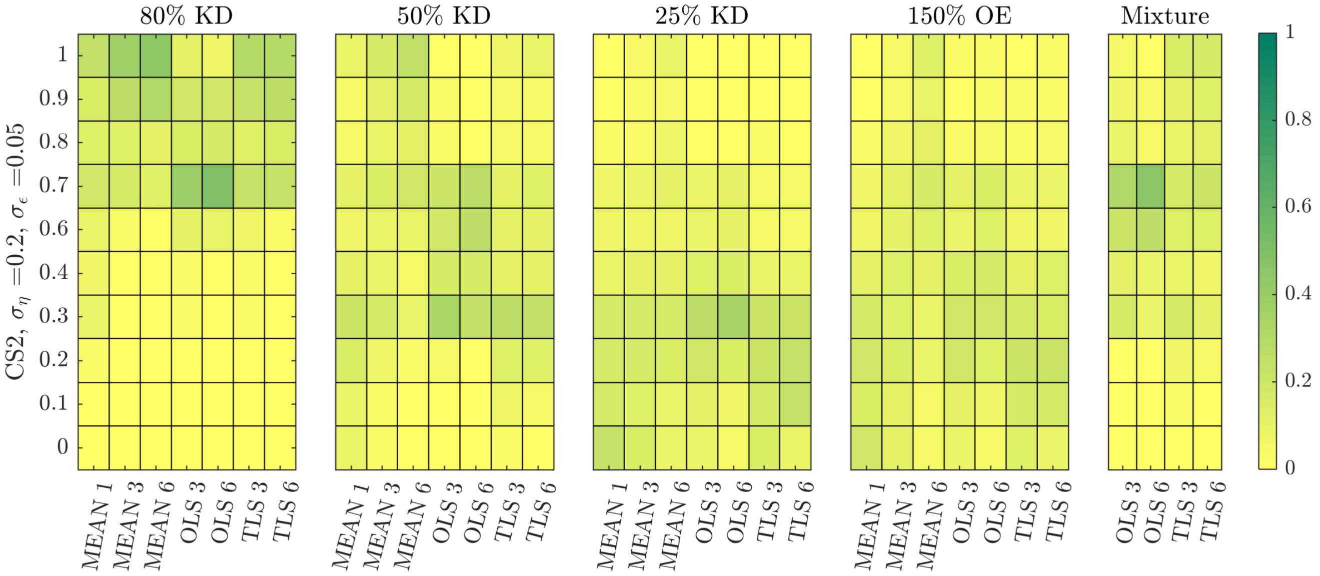

(d) Control strategy 2 and high noise level: $\sigma_\eta = 0.2, \sigma_\varepsilon = 0.05$

Figure S30. (Cont'd) Performance evaluation of all MRA formulations for network reconstruction obtained with the p53 test-bed model. Results obtained with: **(a-b)** intermediate noise level ($\sigma_\eta = 0.1, \sigma_\varepsilon = 0.02$) and **(c-d)** high noise level ($\sigma_\eta = 0.2, \sigma_\varepsilon = 0.05$) for the two different control strategies CS1 and CS2.

Table S3. List of abbreviations

Abbreviation	Meaning	Abbreviation	Meaning
MRA	Modular Response Analysis	LMC	Left Medcouple
LRC	Local Response Coefficient	RMC	Right Medcouple
GRC	Global Response Coefficient	IQR	Interquartile Range
AUC	Area Under the Curve	KD	Knockdown
ROC	Receiver Operating Characteristic	OE	Overexpression
MAPK	Mitogen-Activated Protein Kinase	CS	Control Strategy
ODE	Ordinary Differential Equation	OLS	Ordinary Least Squares
		TLS	Total Least Squares
